# Urban–Wild Interface Diversity: A Comprehensive Checklist of Herpetofauna of Guayaquil, Ecuador

**DOI:** 10.1002/ece3.73504

**Published:** 2026-05-06

**Authors:** Keyko Cruz‐García, Natalia Zapata‐Salvatierra, Andrea E. Narváez, Julián Pérez‐Correa

**Affiliations:** ^1^ Universidad San Francisco de Quito USFQ Instituto de Biodiversidad Tropical IBIOTROP, Laboratorio de Zoología Terrestre, Museo de Zoología Quito Ecuador; ^2^ Museo de Zoología Universidad Técnica Particular de Loja Loja Ecuador; ^3^ Facultad de Ciencias Naturales, Universidad de Guayaquil Ecuador; ^4^ Facultad de Arquitectura e Ingenierías, Universidad Internacional SEK (UISEK) Quito Ecuador; ^5^ Universidad de Especialidades Espíritu Santo Samborondón Ecuador; ^6^ Fundación Great Leaf Quito Ecuador; ^7^ Escuela Superior Politécnica del Litoral, ESPOL, Campus Gustavo Galindo, Facultad de Ciencias de la Vida, Laboratorio de Zoología Guayaquil Ecuador; ^8^ Instituto Nacional de Biodiversidad del Ecuador Quito Ecuador; ^9^ Fundación Para la Conservación e Investigación JaPu Guayaquil Ecuador

**Keywords:** biological invasions, conservation biogeography, Ecuador, Herpetofauna, tropical dry forest, urban biodiversity inventory, urban ecosystem ecology, urban forest remnants

## Abstract

Biodiversity in urban environments is often underestimated, particularly in tropical regions such as Ecuador. This study presents the most comprehensive inventory to date of amphibians and reptiles in Guayaquil, Ecuador's largest and most industrially developed city, based on 17 years (2008–2025) of systematic field surveys, museum records, and literature review. Located on the Pacific coast and adjacent to two global biodiversity hotspots (the Tropical Andes and Tumbes–Chocó–Magdalena), Guayaquil has experienced significant urban expansion, resulting in extensive fragmentation of tropical dry forests and mangrove ecosystems. We document a total of 63 species (19 amphibians, 44 reptiles) occurring within the remnant forest. This inventory includes introduced species, resilient taxa that are adapted to urban environments, and sensitive species that are affected by urban pressure. Notably, species such as *Hemidactylus frenatus* , *Anolis sagrei,* and *Aquarana catesbeiana* thrive in these modified landscapes likely due to behavioral plasticity, rapid reproduction, and tolerance to habitat disturbance, whereas highly sensitive species, including *Alopoglossus festae* and *Lepidoblepharis buchwaldi* , are restricted to less disturbed areas. The study also highlights the presence of rare species with little previous documentation, such as *Atractus microrhynchus* , *Caecilia tenuissima* , *Chironius flavopictus* , and *Drymobius rhombifer* . In addition, expansions in the known distributions of several species in the Guayaquil area were recorded, including *Caiman crocodilus* and *Clelia clelia* , which emphasizes the ecological relevance of this region. Our results underscore the ecological importance of remnant natural habitats within Guayaquil and reveal the need for long‐term monitoring to inform urban biodiversity conservation in rapidly expanding tropical cities.

## Graphical abstract

Amphibians and reptiles in Guayaquil comprise multiple taxonomic orders and occur across conservation areas, forest remnants, and urban parks, including several endemic species and numerous taxa with type localities in the city. Although most species are globally classified as Least Concern, discrepancies between global and national threat assessments indicate that urban populations may face higher localized risks. Together, these patterns underscore the importance of remnant natural habitats within Guayaquil for maintaining coastal herpetofaunal diversity in an increasingly urbanized landscape.

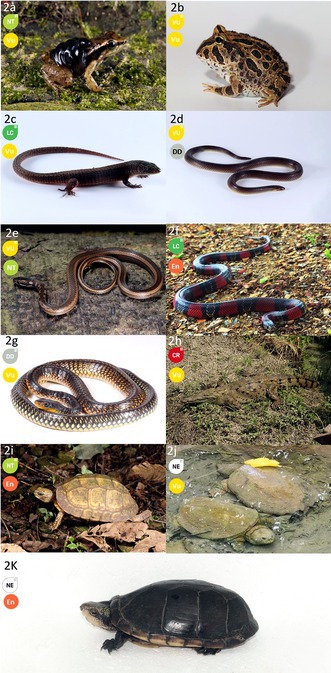

## RESUMEN

La biodiversidad en entornos urbanos usualmente es subestimada, particularmente en regiones tropicales como Ecuador. Este estudio presenta el inventario más completo hasta la fecha de anfibios y reptiles en Guayaquil, la ciudad más grande y desarrollada industrialmente del país, basado en 17 años (2008–2025) de muestreos sistemáticos de campo, registros de museo y revisión de literatura. Ubicada en la costa del Pacífico y adyacente a dos hotspots globales de biodiversidad (los Andes Tropicales y Tumbes–Chocó–Magdalena), Guayaquil ha experimentado una significativa expansión urbana, lo que ha resultado en una extensa fragmentación de los bosques secos tropicales y ecosistemas de manglar. Documentamos un total de 63 especies (19 anfibios y 44 reptiles) presentes dentro de los remanentes de bosque. Este inventario incluye especies introducidas, taxones resilientes adaptados a ambientes urbanos y especies sensibles afectadas por la presión urbana. Notablemente, especies como *Hemidactylus frenatus* , *Anolis sagrei* y *Aquarana catesbeiana* prosperan en estos paisajes modificados, probablemente debido a su plasticidad, rápida reproducción y tolerancia a la perturbación del hábitat; mientras que especies altamente sensibles, como *Alopoglossus festae* y *Lepidoblepharis buchwaldi* , se restringen a áreas menos perturbadas. El estudio también resalta la presencia de especies raras con escasa documentación previa, como *Atractus microrhynchus* , *Caecilia tenuissima* , *Chironius flavopictus* y *Drymobius rhombifer* . Adicionalmente, se registraron ampliaciones en la distribución conocida de varias especies en el área de Guayaquil, incluyendo *Caiman crocodilus* y *Clelia clelia* , lo que enfatiza la relevancia ecológica de esta región. Nuestros resultados subrayan la importancia ecológica de los hábitats naturales remanentes dentro de Guayaquil y destacan la necesidad de monitoreo a largo plazo para informar estrategias de conservación de la biodiversidad urbana en ciudades tropicales en rápida expansión.

## 1 Introduction

Ecuador boasts a remarkable diversity of habitats and ecosystems, shaped by its equatorial location, the varied mountain ranges, multiple climate zones, and ocean current patterns (Cuesta et al. 2017). On the other hand, the country lies between two of the five biodiversity hotspots in South America: the tropical Andes and the Tumbes–Chocó–Magdalena Hotspot (Myers et al. 2000; Mestanza‐Ramón et al. 2023). This geographic positioning not only supports extraordinary biodiversity but also fosters high levels of endemism (Castro et al. 2023).

One notable example is the Chongón‐Colonche Cordillera, a mountain range on the Pacific coast that lies fewer than 20 km from the shoreline and over 200 km from the main Andean ranges (Jadán et al. 2022). It extends across three Ecuadorian provinces: Guayas, Manabí, and Santa Elena (Salvatierra et al. 2010). Despite its proximity to major urban centers, long‐term, integrative datasets on herpetofauna in this region remain fragmented or unpublished, and even fewer studies address the conservation of these critical taxa (Salvatierra et al. 2010; Navarrete et al. 2011; Reyes‐Puig et al. 2017).

Amphibians and reptiles are distributed unevenly across global ecoregions (Brum et al. 2022), due to ecological, geographic, and evolutionary factors (Roll et al. 2017) with the highest concentrations in tropical regions (Gumbs et al. 2020). Their distributions are increasingly shaped by human‐induced landscape changes, such as habitat fragmentation, pollution, and environmental stress linked to urbanization (e.g., Salvador et al. 2017; De Solan et al. 2018; Cordier et al. 2021; Brum et al. 2022; Shacham et al. 2023). While some species successfully adapt to urban conditions, others fail to cope, leading to restricted distributions confined to native habitats. Species highly sensitive to urban expansion suffer disproportionately from the loss of green spaces, limited dispersal capabilities, and dependence on clean water resources (Hutto and Barrett 2021).

Urbanization is an accelerating, often irreversible process that leaves few spaces for native vegetation (Dearborn and Kark 2010). However, the sustainability of urban environments depends precisely on preserving natural spaces within and around cities (Cicea and Pirlogea 2011). These areas serve as reservoirs of organic matter, critical water sources, and mitigate the urban heat island effect, improving air quality and influencing local microclimates (Myers et al. 2000). Furthermore, remnant forests often provide the most accessible natural and semi‐natural habitats for urban fauna, acting as essential refuges for biodiversity such as amphibians and reptiles.

As Ecuador's largest city and principal port, Guayaquil exemplifies the challenges of uneven urban expansion and natural forest fragmentation (Peek et al. 2017). It is Ecuador's most economically significant city (Swyngedouw 1997), yet it faces urban, environmental, and social degradation due to neglect management (Delgado 2013). Only 37% of Guayaquil's urban area is covered by remnants of native vegetation, which safeguard local wildlife (Camacho et al. 2011; Magle et al. 2012), although geographic calculations reflect only 23% of these areas remaining, as calculated by one of us (J.P.C.). The city hosts two distinct ecosystems: the Tropical Dry Forest, associated with the Chongón‐Colonche Cordillera that crosses the city, and the mangrove ecosystems linked to the Guayas River estuary (Ordóñez 2020). Both ecosystems are threatened by activities such as open‐pit limestone mining in the Chongón‐Colonche Mountain range and extensive aquaculture in mangrove areas. These issues are evident in two significant protected areas: Cerro Blanco Protective Forest, one of the last dry forest remnants on Ecuador's coast, and El Salado Mangrove Wildlife Production Reserve, one of the country's largest coastal marine reserves.

Despite Guayaquil's rapid urban growth and land‐use changes, the city continues to harbor diverse wildlife, much of which goes unnoticed within its urban perimeter (Amador‐Oyola 2015). Historically, cities like Guayaquil have been considered low in biodiversity; however, other studies reveal that urban areas safeguard a substantial richness (Alvey 2006). In Guayas province, where Guayaquil is located, numerous studies (e.g., Almendáriz and Carr 1992, 2012; Salvatierra et al. 2010; Amador and Martínez 2011; Amador et al. 2017; Bustamante and Alava 2020; Cuadrado et al. 2020; Cruz‐García et al. 2020, 2023; Narváez et al. 2020; Ramon et al. 2022; Cruz‐García and Zapata‐Salvatierra 2024) highlight the significant biodiversity of the Chongón‐Colonche Cordillera and estuaries ecosystems. These studies underscore the importance of preserving the remaining native vegetation to foster urban resilience. While previous studies have documented aspects of Guayaquil's herpetofauna, including recent research by Moretta‐Urdiales et al. (2025), the biodiversity of the urban landscape has yet to be comprehensively assessed. This manuscript presents the most extensive inventory to date of amphibians and reptiles in Guayaquil's remnant forests and urban gardens, based on 17 years of data collection dedicated to understanding herpetofauna and their habitats.

## 2 Materials and Methods

### 2.1 Study Area

**FIGURE 1:**
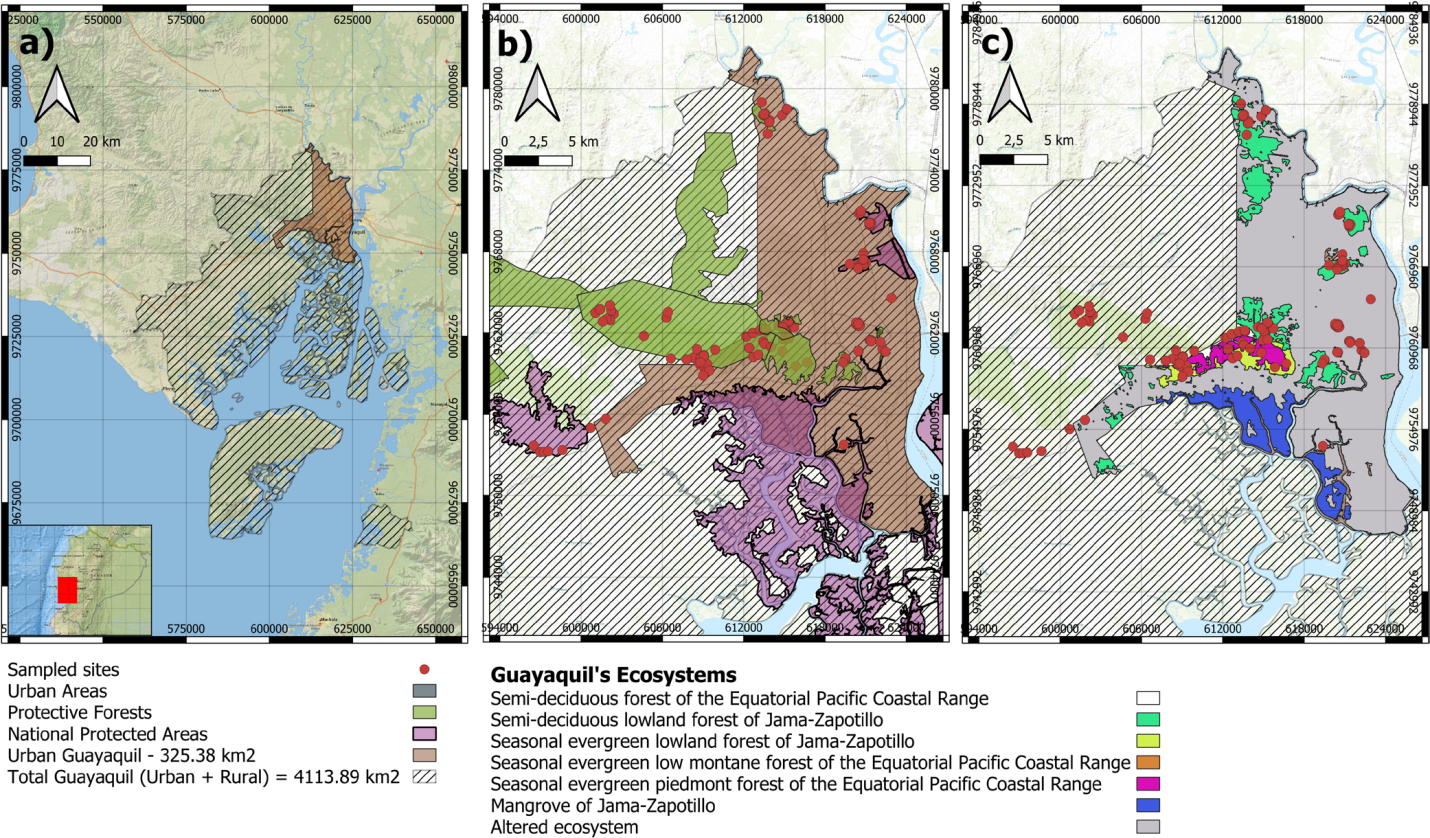
Study area in Guayaquil, Ecuador. Panel (a) shows the spatial extent of the study area, distinguishing between urban and rural zones. Panel (b) depicts the distribution of conservation areas, including protective forests and national protected areas. Panel (c) illustrates the spatial distribution of remnant native ecosystems and altered areas within the urban territory, together with the locations of sampled sites.

Guayaquil is located on the northern side of the Gulf of Guayaquil, in the province of Guayas, at an elevation of 4.6 m above sea level (m.a.s.l.) along the Pacific Coast in southwestern Ecuador. The city lies within a transitional zone influenced by its proximity to the Chongón‐Colonche Cordillera, a low mountain range that extends parallel to the coastline. This topography contributes to habitat heterogeneity, ranging from dry forests to wetland areas. Guayaquil's urban core is interspersed with remnant natural patches, including protected areas such as the Cerro Blanco Protective Forest, Prosperina Protective Forest and Vegetation, El Salado Mangrove Wildlife Production Reserve, and Samanes National Recreation Area, which serve as biodiversity refugia among urban expansion. Guayaquil's total area is 4113.89 km^2^ meanwhile urban area corresponds to 7.91% of total Guayaquil territory with an extension of 325.38 km^2^ where 75.21 km^2^ correspond to native vegetation (Table 1, Figure 1).

**TABLE 1:** Spatial extent of urban Guayaquil, native ecosystems, and protected areas within Guayaquil, Ecuador.

General	Guayaquil (km^2^)	Urban Guayaquil (km^2^)	Percentage
**Total area**	4113.89	325.38	7.91%
**Native area**	2274.89	75.21	3.31%
**Ecosystems**			
Lowland semideciduous Forest of Jama‐Zapotillo	388.46	33.97	10.44%
Lowland seasonal evergreen forest of Jama‐Zapotillo	10.5	7.19	2.21%
Mangrove of Jama‐Zapotillo	679.12	23.50	7.22%
Semideciduous forest of the coastal range of the Ecuadorian Pacific	29.55	1.79	0.55%
Low montane seasonal evergreen forest of the coastal range of the Ecuadorian Pacific	0.086	0.04	0.01%
Piedmont seasonal evergreen forest of the coastal range of the Ecuadorian Pacific	33.17	8.72	2.68%
Lowland deciduous forest and shrubland of Jama‐Zapotillo	119.93	0.00	0.00%
Deciduous forest of the coastal range of the Ecuadorian Pacific	0.7	0.00	0.00%
Lowland deciduous forest of Jama‐Zapotillo	1013.41	0.00	0.00%
**Protective forest**			
Subcuenca del Río Chongón	169.91	0	0.00%
Bosque y Vegetación Protectora Cerro Blanco	59.59	7.49	2.30%
Bosque y Vegetación Protector Papagayo de Guayaquil	36.64	0.28	0.09%
Bosque y Vegetación Protector Bosqueira	1.31	1.29	0.40%
Bosque y Vegetación Protector Prosperina	2.43	1.43	0.44%
Bosque y Vegetación Protectora Cerro Paraíso	2.98	2.98	0.92%
Bosque y Vegetación Protector Cerro Azul	8.80	8.80	2.70%
Bosque y Vegetación Protectora El Sendero Palo Santo	0.04	0.04	0.01%
Reserva de Producción de Fauna “Manglares El Salado”	0.47	0.47	0.14%
Yasún Limbo	40.15	0	0.00%
Los Gelices	11.23	0	0.00%
**National protected areas**			
**Parque Lago national recreation area**	21.5	0	0.00%
**Los Samanes national recreation area**	8.52	8.52	2.62%
**El Salado mangrove wildlife production reserve**	155.42	28.46	8.75%
**Wildlife Refuge Manglares EL Morro**	353.94	0	0.00%

*Note:* Values indicate total area at the city scale, area within the urban perimeter, and the percentage of each category occurring within urban Guayaquil.

Estimates of native vegetation cover within the urban perimeter, as well as land‐use change between 2008 and 2022, were derived from a geographic information system (GIS) analysis conducted by the authors. The analysis used official ecosystem classification layers and land‐use layers retrieved from the Ministry of Environment and Energy interactive platform (http://ide.ambiente.gob.ec/mapainteractivo/), together with urban boundary shapefiles projected in WGS84 and analyzed in QGIS (version 3.40.14‐Bratislava). Spatial analyses were performed to quantify the extent of remnant native ecosystems within the current urban footprint. While these calculations provide an updated approximation of vegetation cover, they are presented as estimates and should be interpreted in the context of ongoing land‐use change.

According to Ecuador's ecosystem classification (Ministerio del Ambiente del Ecuador 2012), five ecosystem types are represented within urban Guayaquil, including two national protected areas and seven protective forests (Table 1; Figure 1). Climatically, Guayaquil experiences a tropical savanna climate (Aw) under the Köppen classification, characterized by a wet season from January to May and a dry season from June to December (Peel et al. 2007). These seasonal patterns strongly influence the availability of water and microhabitats, shaping the distribution and activity of the city's herpetofauna, particularly during the dry season.

**FIGURE 2:**
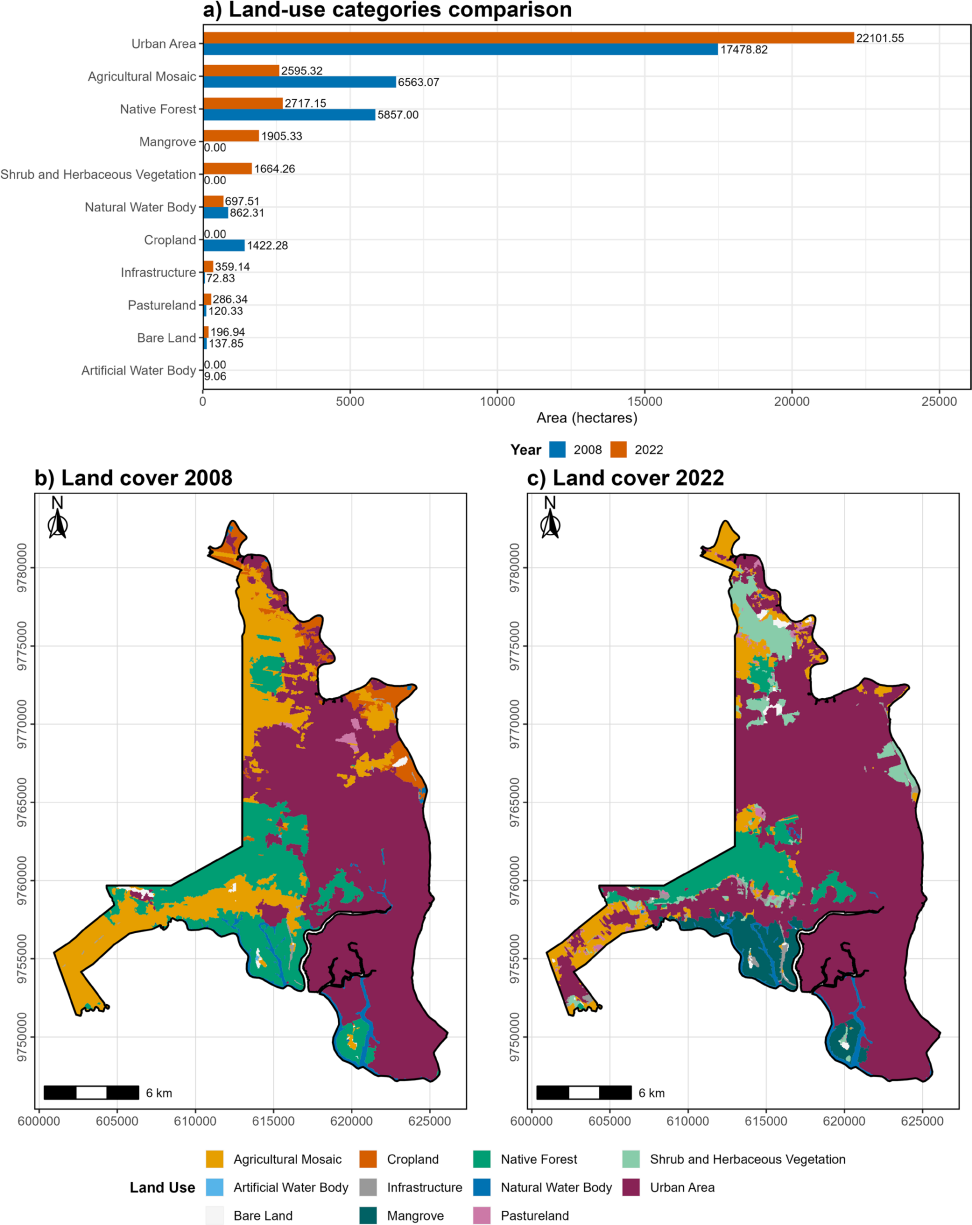
Land‐use change in urban Guayaquil between 2008 and 2022. Panel (a) shows the quantitative comparison of land‐use categories (area in hectares) between 2008 and 2022. Panel (b) presents the spatial distribution of land‐use classes in 2008, and panel (c) the corresponding distribution in 2022. Together, these panels illustrate the expansion of urban areas and the reduction of native ecosystems over time.

Land‐use changes between 2008 and 2022 (the most recent available dataset) indicate a marked expansion of urban area from 17,478.62 to 22,101.55 ha (26.4% increase), accompanied by a substantial reduction in native forest cover from 5857 to 2717.15 ha (Figure 2). These changes reflect a clear trend of urban incursion into natural ecosystems within the study area.

### 2.2 Survey Methodology

Amphibians and reptiles were recorded through surveys conducted between January 2008 and February 2025 by some of the authors (KCG, AEN, NZS). Field collections followed ethical and legal protocols, with specimens deposited in Ecuadorian museum collections: Museo de Zoología de la Universidad del Azuay (MZUA), Museo de Zoología Universidad San Francisco de Quito (ZSFQ), Museo de Zoología, Universidad Técnica Particular de Loja (MUTPL), Museo de Zoología de la Pontificia Universidad Católica del Ecuador (QCAZ), and Museo de la Escuela Politécnica Nacional (MEPN). Specimens’ preparation follows standard preservation protocols; individuals were captured by hand, photographed, euthanized using 2% roxicaine (an anesthetic), then fixed in 10% formalin and preserved in 70% ethanol. Liver and muscle tissue were obtained before fixation and preserved in 90% ethanol. Collection permits were approved by the Ministerio del Medio Ambiente, Agua y Transición Ecológica del Ecuador (Permit Nos. MAE‐UAF‐DPAG‐20218‐2522‐E, MAAE‐DBI‐CM‐2022‐0231, MAAE‐ARSFC‐2022‐2058, MAAE‐DBI‐DBI‐CM‐2022‐0222, MAATE‐ARSFC‐2023‐0063, MAATE‐ARSFC‐2024‐1019). Fieldwork focused on 11 remnant forests (including private and national reserves) and five revegetated parks within the urban area (Table 2).

**TABLE 2:** Sampled areas subcategorized into conservation and nonconservation areas.

Name	Type
Bosque y Vegetación Protector Bosqueira	Conservation area
Bosque y Vegetación Protector Cerro Blanco^a^	Conservation area
Bosque y Vegetación Protector Cerro Blanco (507)^b^	Conservation area
Bosque y Vegetación Protector Cerro Paraíso	Conservation area
Bosque y Vegetación Protector El Sendero Palo Santo	Conservation area
Bosque y Vegetación Protector Prosperina	Conservation area
Bosque y Vegetación Protectora Cerro Colorado^c^	Conservation area
Bosque y Vegetación Protector Cerro Azul	Conservation area
Jardín Botánico de Guayaquil^c^	Urban park
Los Samanes National Recreation Area	Conservation area
Parque Lago National Recreation Area^a^	Conservation area
El Salado Mangrove Wildlife Production Reserve	Conservation area
Parroquia Urbana Chongón, Guayaquil	Urban park
Zoológico El Pantanal	Urban park
Natural Science Faculty – Guayaquil University	Remnant
Northern Urban Guayaquil	Urban park
Southern Urban Guayaquil	Urban park

*Note:* Remnant category corresponds to native vegetation not listed under any protection status. Urban parks correspond to parks with some or no native vegetation but with significant habitat to support herpetofauna.

^a^ Correspond to areas very close to urban boundaries that are worth including in the analysis.

^b^ Cerro Blanco (507) corresponds to the section of Cerro Blanco included exclusively in the urban area.

^c^ Cerro Colorado and Jardín Botánico are areas encompassed within Los Samanes National Recreation Area but with different protection activities.

Records were supplemented with data from Ecuadorian museum collections: MZUA, ZSFQ, MUTPL, QCAZ and MEPN. We also included online database records from Bioweb Ecuador (Ron et al. 2024; Torres‐Carvajal et al. 2025), the National Institute of Biodiversity (INABIO) and the Global Biodiversity Information Facility (GBIF). Rural parishes were excluded from this study due to their geographical distance from Guayaquil's urban boundaries. We also included historical records gathered through an extensive scientific literature review. Additional information was obtained from gray literature, images published in newspapers, local websites, and social media platforms.

### 2.3 Data Processing

The information used in this study integrates primary records obtained during fieldwork, as well as secondary data from scientific literature and other documented sources, which were compiled, standardized, and jointly processed. This approach allowed for the construction of a robust dataset that reflects both the evidence generated by the authors and the existing knowledge for the study area.

The primary data, obtained directly within the framework of this study, include detailed information on the specimens collected and examined by the researchers. These data comprises geographic coordinates, collection dates, sex, developmental stage, and museum catalog numbers, and is presented systematically in Appendix S1 to ensure the traceability and reproducibility of the original records.

Additionally, the secondary information was compiled from scientific publications and other sources and subjected to a critical appraisal process before inclusion. Only records supported by verifiable information were considered, excluding those lacking clear identification criteria, sufficient geographic precision, or support in recognized scientific collections. Historical observations that have not been confirmed in recent decades were omitted to reduce the inclusion of potentially outdated or unreliable records. Data from nonconventional sources were carefully reviewed and records whose validity could not be independently verified were discarded to ensure the consistency of the final dataset. Occurrence information for Guayaquil, associated museum codes, and records from external sources are presented in consolidated form in Appendix S2.

The final species list was standardized following the taxonomic frameworks currently accepted by Amphibian Species of the World (Frost 2025) and The Reptile Database (Uetz et al. 2025). The conservation status assessments were based on the IUCN (2025), as well as on regional lists for amphibians (Ortega‐Andrade et al. 2021) and reptiles (Carrillo et al. 2005). In addition, the inclusion of each species in the appendices of the Convention on International Trade in Endangered Species of Wild Fauna and Flora (CITES 2020) was reviewed to document their international regulatory status, and this information was incorporated into the corresponding tables.

## 3 Results

Field studies conducted between 2008 and 2025 documented a total of 63 herpetofauna species in Guayaquil, including 19 amphibians and 44 reptiles, recorded in tropical dry forest remnants, conservation areas, and urban environments. The dataset integrates records obtained directly during fieldwork with verified historical records from national and international scientific collections, which are summarized in Appendix S1. Species records span a broad temporal range and multiple habitat types within the urban and peri‐urban landscape.

Amphibians were represented by two orders, Anura and Gymnophiona, with Anura comprising the majority of recorded species. Amphibian records were primarily associated with conservation areas (19 spp.) and forest remnants (17 spp.), although several species were also documented in urban parks (5 spp.). Six amphibian species recorded are endemic to Ecuador, and three taxa have type localities in Guayaquil (Table 3).

**TABLE 3:** Amphibian species recorded in Guayaquil according to area type: Conservation areas (CA), urban parks (UP), and remnant native vegetation (REM). *Type locality* indicates species for which Guayaquil represents the type locality.

Taxa	Area by disturbance	Type locality	IUCN	ERL	Appendix Fig.
**Order Anura**					
**Bufonidae**					
*Rhinella bella*	CA, UP, REM		NE	NE	3.1
**Ceratophryidae**					
*Ceratophrys stolzmanni*	CA, REM		VU	VU	3.2
**Craugastoridae**					
*Craugastor longirostris*	CA, REM		LC	LC	3.3
**Dendrobatidae**					
Epipedobates machalilla*	CA, REM		LC	LC	3.4
Hyloxalus infraguttatus*	CA, REM		NT	VU	3.5
**Hylidae**					
*Boana rosenbergi*	CA		LC	LC	3.6
*Scinax quinquefasciatus*	CA, UP, REM		LC	LC	3.7
*Smilisca phaeota*	CA, REM		LC	LC	3.8
*Trachycephalus jordani*	CA, UP, REM		LC	LC	3.9
Trachycephalus quadrangulum*	CA, REM	X	LC	LC	3.10
**Leptodactylidae**					
*Engystomops pustulatus*	CA, UP, REM	X	LC	LC	3.11
Engystomops randi*	CA, REM		LC	LC	3.12
*Leptodactylus labrosus*	CA, UP, REM		LC	LC	3.13
*Leptodactylus melanonotus*	CA, REM		LC	LC	3.14
*Leptodactylus ventrimaculatus*	CA, REM		LC	LC	3.15
**Ranidae**					
*Aquarana catesbeiana*	CA, REM		LC	NE	3.16
**Strabomantidae**					
Barycholos pulcher*	CA, REM		LC	LC	3.17
*Pristimantis achatinus*	CA, REM		LC	LC	3.18
**Order Gymnophiona**					
**Caeciliidae**					
Caecilia tenuissima*	CA	X	DD	EN	3.19

*Note:* Conservation status follows the IUCN Red List and the Ecuadorian Red List (ERL). *Appendix Fig*. refers to the corresponding photograph in Appendix S3. Species with * are Endemic to Ecuador.

Reptiles were represented by four orders, with Squamata accounting for most of the recorded species. Reptile presence was documented across all habitat categories, including conservation areas (42 spp.), forest remnants (34 spp.), and urban parks (9 spp.). Three reptile species recorded in this study are endemic to Ecuador, and 11 have Guayaquil as the type locality (Table 4).

**TABLE 4:** Reptile species recorded in Guayaquil according to area type: Conservation areas (CA), urban parks (UP), and remnant native vegetation (REM).

Taxa	Area type	Type locality	IUCN	ERL	CITES	Appendix Fig.
**Order Crocodylia**						
**Alligatoridae**						
*Caiman crocodilus*	CA		LC	LC	II	4.1
**Crocodylidae**						
*Crocodylus acutus*	CA, REM		CR	VU		4.2
**Order Squamata: Amphisbaenia**						
**Amphisbaenidae**						
*Amphisbaena fuliginosa varia*	CA		NE	NT		4.3
**Order Squamata: Sauria**						
**Anolidae**						
*Anolis binotatus*	CA, REM	X	LC	DD		4.4
*Anolis festae*	CA, REM		NT	NT		4.5
*Anolis gracilipes*	CA, UP, REM		LC	LC		4.6
*Anolis sagrei*	UP, CA		LC	NE		4.7
**Alopoglossidae**						
*Alopoglossus harrisi*	CA, REM		LC	VU		4.8
**Gekkonidae**						
*Hemidactylus frenatus*	CA, UP, REM		LC	NE		4.9
**Iguanidae**						
*Iguana iguana*	CA, UP, REM		LC	LC	II	4.10
**Phyllodactylidae**						
*Phyllodactylus reissii*	CA, UP, REM	X	LC	LC		4.11
**Polychrotidae**						
*Polychrus femoralis*	CA, REM	X	LC	NT		4.12
**Spheaerodactylidae**						
*Gonatodes caudiscutatus*	CA, UP, REM		LC	LC		4.13
Lepidoblepharis buchwaldi*	CA, REM		LC	NT		4.14
**Teiidae**						
*Holcosus septemlineatus*	CA, REM		LC	LC		4.15
*Medopheos edracanthus*	CA, REM		LC	LC		4.16
**Tropiduridae**						
*Stenocercus iridescens*	CA, REM		LC	LC		4.17
**Order Squamata: Serpentes**						
**Boidae**						
*Boa imperator*	CA, UP, REM		LC	VU	II	4.18
**Colubridae**						
*Atractus microrhynchus*	CA	X	VU	DD		4.19
*Chironius flavopictus*	CA	X	DD	VU		4.20
*Clelia clelia*	CA		LC	LC		4.21
*Coniophanes dromiciformis*	CA, REM	X	VU	NT		4.22
*Dendrophidion brunneum*	CA, REM	X	LC	NT		4.23
Dipsas georgejetti*	CA		NE	NE		4.24
*Drymarchon melanurus*	CA, REM		LC	NT		4.25
*Drymobius rhombifer*	CA		LC	LC		4.26
*Imantodes cenchoa*	CA, REM		LC	LC		4.27
*Lampropeltis micropholis*	CA, REM		LC	EN		4.28
*Leptodeira ornata*	CA, REM		LC	LC		4.29
*Leptophis occidentalis*	CA, REM	X	NE	NE		4.30
*Mastigodryas pulchriceps*	CA, REM		LC	NT		4.31
*Mastigodryas reticulatus*	CA, REM	X	NT	NT		4.32
Oxybelis transandinus*	CA, REM	X	NE	NE		4.33
*Oxyrhopus petolarius*	CA, REM		LC	LC		4.34
*Stenorrhina degenhardtii*	CA, REM		LC	NT		4.35
*Tantilla capistrata*	CA, REM		LC	DD		4.36
**Elapidae**						
*Micrurus bocourti*	CA, REM		LC	VU		4.37
*Micrurus mipartitus*	CA, REM		LC	LC		4.38
**Leptotyphlopidae**						
*Epictia subcrotilla*	CA, REM		LC	DD		4.39
**Typhlopidae**						
*Indotyphlops braminus*	UP		LC	NE		4.40
**Viperidae**						
*Bothrops asper*	CA, UP, REM		LC	LC		4.41
**Order Testudines**						
**Chelydridae**						
*Chelydra acutirostris*	CA, UP, REM	X	NE	VU		4.42
**Geoemydidae**						
*Rhinoclemmys annulata*	CA, REM		NT	EN		4.43
**Kinosternidae**						
*Kinosternon leucostomum*	CA		NE	EN		4.44

*Note:* *Type locality* indicates species for which Guayaquil represents the type locality. Conservation status follows the IUCN Red List and the Ecuadorian Red List (ERL), and CITES appendices are indicated where applicable. *Appendix Fig*. refers to the corresponding photograph in Appendix S4. Species with * are Endemic to Ecuador.

**FIGURE 3:**
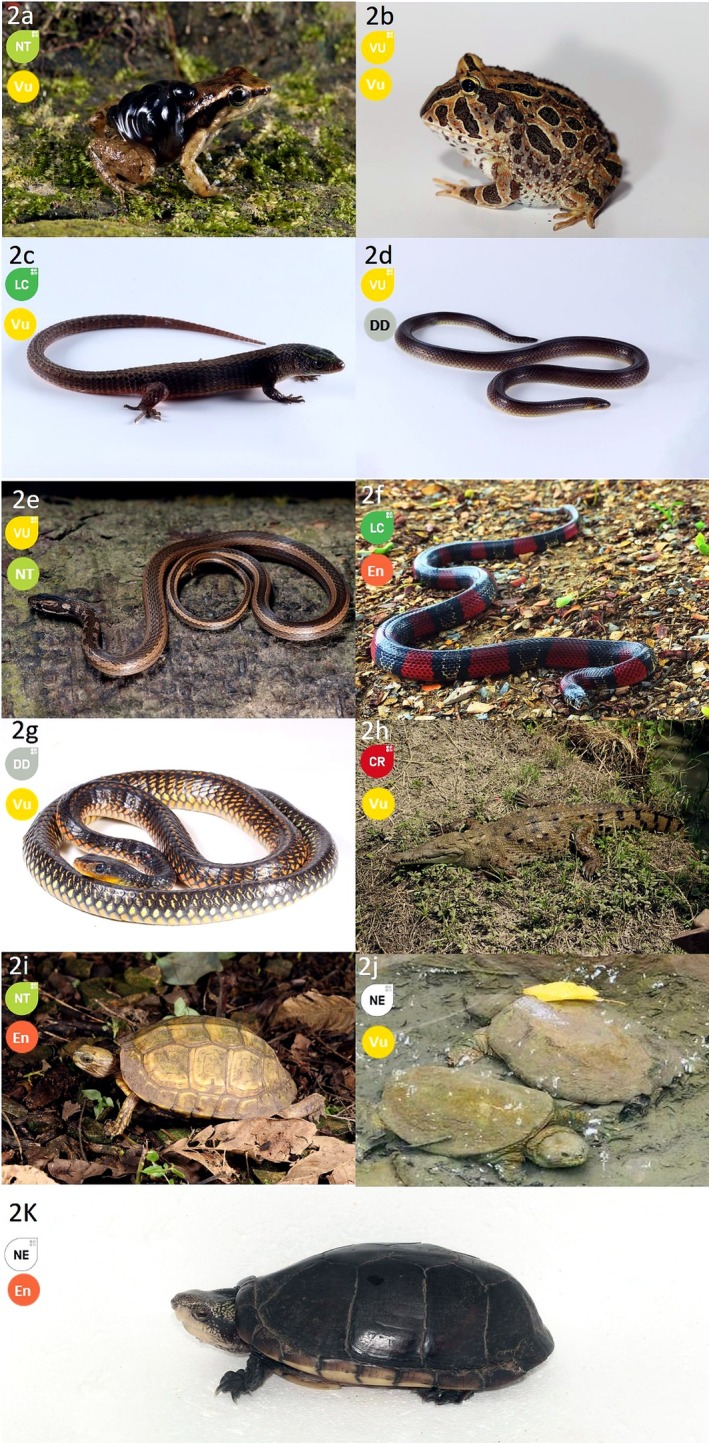
Representative amphibian and reptile species of conservation concern recorded in remnant urban habitats of Guayaquil, Ecuador. The upper badge in each panel indicates the global conservation status according to the IUCN Red List of Threatened Species, whereas the lower badge indicates the national conservation status according to the Ecuadorian Red List. National categories follow Ortega‐Andrade et al. (2021) for amphibians and Carrillo et al. (2005) for reptiles. CR, critically endangered; DD, data deficient; EN, endangered; LC, least concern; NE, not evaluated; NT, near threatened; VU, vulnerable.

Based on global assessments (IUCN 2025), the recorded herpetofauna are classified as follows: seven Not Evaluated (NE), two Data Deficient (DD), 46 Least Concern (LC), four Near Threatened (NT), three Vulnerable (VU), and one Critically Endangered (CR). In contrast, several species are assigned to higher threat categories in national Red Lists, for example: *Lampropeltis micropholis* , *Rhinoclemmys annulate*, and *Kinosternon leucostomum* are considered Endangered (En) in Ecuador (Carrillo et al. 2005; Ortega‐Andrade et al. 2021), highlighting differences between global and national conservation assessments. Together, these results highlight Guayaquil as a significant reservoir of coastal herpetofaunal diversity despite intense urbanization (Figure 3).

Given the extensive number of species recorded and the level of detail associated with each record, comprehensive taxonomic accounts are provided as Supporting Information. Appendix S1 includes detailed species accounts for all recorded taxa, incorporating material examined, geographic coordinates, elevation, dates, collectors or observers, and voucher or observation information. Appendix S2 presents the complete checklist of amphibians and reptiles recorded in Guayaquil, including taxonomic classification and conservation status. Visual documentation of representative species is provided in Appendix S3 (amphibians) and Appendix S4 (reptiles), facilitating taxonomic verification and supporting species identification.

## 4 Discussion

This study represents the most comprehensive inventory of amphibians and reptiles for Guayaquil to date, documenting 63 species (19 amphibians and 44 reptiles) found across tropical dry forest in 11 remnant and five urban areas. These findings demonstrate the resilience of herpetofauna species in urban ecosystems while simultaneously emphasizing the persistent challenges posed by habitat loss, habitat fragmentation, habitat degradation, urban expansion, and pollution. These pressures are particularly acute in rapidly growing cities like Guayaquil, which face high levels of environmental disturbance (Patiño‐Aroca et al. 2024).

### 4.1 A Decade of Progress in Herpetofauna Records

There have been notable advances since the last comprehensive inventory in 2015 (Amador‐Oyola 2015; Cornejo 2015). In a more recent inventory, Ramon et al. (2022) reported 13 reptiles and 12 amphibians in a single remnant forest (Prosperina Protective Forest and Vegetation), while Cruz‐García and Zapata‐Salvatierra (2024) documented in total 20 amphibians and 37 reptiles for Cerro Blanco Protective Forest. We highlight the recent additions to the Guayaquil checklist species, such as the common house gecko ( *Hemidactylus frenatus* ), brown anole ( *Anolis sagrei* ), the American bullfrog (*Aquarana catesbeiana*), the Flowerpot Blindsnake ( *Indotyphlops braminus* ), all of which are alien invasive species (Amador et al. 2017; Narváez et al. 2023, 2024; Zavala and Arteaga 2025).

Although *Indotyphlops braminus* was recently reported for Guayaquil (Zavala and Arteaga 2025), all previous records were based solely on citizen science observations and the authors' observations. The record documented in this work constitutes the first formal confirmation of the species in the locality through a specimen collection, providing a verifiable and solid basis for taxonomic identification. Furthermore, this study contributes to the knowledge of the region by reporting for the first time the presence of *Caiman crocodilus* in Guayaquil and its surroundings. Although this newly recorded species was not physically collected, photographs and detailed taxonomic analysis of specific characters confirmed its identification and ensured that there were no potential misidentifications. These additions not only expand the regional checklist but also highlight the necessity of incorporating photographic records with detailed taxonomic analyses to fill knowledge gaps in terms of biodiversity reports. Similarly, the case of another introduced species further points to the importance of formal verification of records in the region. A specimen of *Xenopus laevis* was recorded on June 1, 2006, on the banks of the Babahoyo River, Samborondón canton, and subsequently deposited in the collection of the Museo de Zoología Universidad San Francisco de Quito under the code ZSFQ4320 (Diego F. Cisneros‐Heredia, pers. Comm.). This species, commonly traded as an aquatic pet in several Latin American countries (IUCN SSC Amphibian Specialist Group 2020), may have been introduced through this practice. The reported site is located near Guayaquil, as the Babahoyo River is a tributary of the Guayas River, which flows through the city. This is the only known record of *Xenopus* in Guayaquil surroundings. Overall, our study reports a remarkable 50% increase in amphibian and reptile species for Guayaquil, reflecting the effort made to characterize its herpetofauna and expand sampling areas over the past 15 years. This trend highlights the importance of collaborative and continuous monitoring toward the understanding of the dynamics of biodiversity in urban and peri‐urban landscapes.

Among the species recorded, 12 have Guayaquil and its surroundings as their type locality. However, several species—such as *Ninia schmidti* (Jan 1862), *Atractus elaps* (Günther 1858), and *Anolis fasciatus* (Boulenger 1885), among others—are listed with Guayaquil as their type locality but were not detected during our surveys. It is possible that the specimens used for their original descriptions were transported to Guayaquil via maritime trade rather than being native to the region. Alternatively, urban expansion, habitat degradation, and increasing anthropogenic pressures may have led to the local extirpation of some species, as may be the case for *Tropidophis gularis* (Peters 1860) and *Epictia guayaquilensis* (Orejas‐Miranda and Peters 1970), which have not been observed since their original descriptions.

### 4.2 Resilience Among Urbanization

Guayaquil harbors a remarkable portion of the herpetofauna diversity of Ecuador's coastal region, where 126 amphibian species (Ron et al. 2024) and 182 reptile species (Torres‐Carvajal et al. 2025) have been recorded. This city safeguards 63 species, including 19 amphibians and 44 reptiles, occurring in remnants of tropical dry forest and urban environments. This richness represents approximately 40% of the amphibians and 50% of the reptiles reported for Guayas Province, which hosts a total of 135 species, 47 amphibians and 88 reptiles (Garzón‐Santomaro et al. 2023). The persistence of this biodiversity is particularly significant, considering the high level of urbanization, the rapid loss of native vegetation cover (Horstman 2012), and the elevated levels of air, light, and noise pollution that characterize Guayaquil (Calero‐Amores et al. 2017; Velastegui‐Montoya et al. 2023; Patiño‐Aroca et al. 2024), highlighting the presence of ecologically valuable and functionally active habitats within this urban matrix.

Although these taxa are known for their high sensitivity to landscape disturbance and pollution (Roa‐Rivera 2020; Costa et al. 2024; Jacob et al. 2024), some of the species, here reported, can be considered resilient as they have coped with urbanization. For example, species such as *H. frenatus* , *I. braminus, G. caudiscutatus*, *P. reissi*, *A. sagrei* , *R. bella, S* *. quinquefasciatus* , *T* *. jordani* , and *E. pustulatus* can thrive in these environments (MECN 2010; Narváez et al. 2024; Zavala and Arteaga 2025) due to their adaptations to urban habitats related to traits like rapid reproduction and behavioral plasticity. For instance, *H. frenatus* establishes quickly; females can store viable sperm for up to 54 weeks (Yamamoto and Ota 2006) and have up to three clutches per year (McKay and Milenkaya 2020). In fact, two of the aforementioned species ( *H. frenatus* and *A. sagrei* ) are introduced and are relatively more abundant in comparison to native species (Torres‐Carvajal and Tapia 2011; Torres‐Carvajal 2015; Mora and Salas 2025; Narváez et al. 2024). Guayaquil's role as Ecuador's primary port increases the risk of new exotic species introduction via maritime transport, a factor recognized as the main route of introduction for these species (Bouda et al. 2017; O'Brien et al. 2017). The four aforementioned amphibians’ species have also been recorded in urban parks and have demonstrated adaptive capacity, despite of limited habitat availability and having been reported with incidences of *Batrachochytrium dendrobatidis* (Bd) (Moretta‐Urdiales et al. 2025). Consequently, strengthening monitoring programs at port facilities and along trade routes is essential to mitigate this threat.

### 4.3 Species Loss and Rarity

Certain species reported in earlier studies have become particularly rare or locally absent. For example, *Atractus microrhynchus* , described by Cope in 1868, has not been observed since its original description in Guayaquil, and its type specimen is missing (Passos et al. 2012). In a parallel case, *Caecilia tenuissima* has been recorded only once in the Cerro Blanco Protective Forest since its discovery in 1973 (Taylor 1973); its reappearance in 2021 after five decades confirms the species’ persistence, underscoring the importance of long‐term monitoring. A similar case is that of *Drymobius rhombifer* , recorded only in two collections made in Guayaquil in 1881 (Orrell 2025). Due to the lack of subsequent records in the region, it has been speculated that the collection locality could be erroneous. However, the recent finding validates the presence of the species in the city and its surroundings, expanding its known distribution. Also, it is important to highlight that *Corallus blombergi*, a threatened species, has been historically reported from the city of Guayaquil (Henderson et al. 2001); however, it has not been observed again. This absence underscores the need for long‐term monitoring programs and the strengthening of conservation efforts focused on herpetofauna in urban and peri‐urban environments.

Species such as *Anolis festae, Ceratophrys stolzmanni, Coniophanes dromiciformis, Hyloxalus infraguttatus, and Mastigodryas reticulatus* remain threatened mainly by habitat loss, agricultural expansion, and urbanization. Meanwhile, species like *Crocodylus acutus* and *Rhinoclemmys annulata* face additional threats from poaching and habitat degradation (IUCN 2025). Conservation efforts should prioritize these taxa, alongside research on poorly understood subterranean species like *Caecilia tenuissima* , *Amphisbaena fuliginosa varia* , and *Epictia subcrotilla* , which are challenging to detect and to estimate populations due to their fossorial habits (Adalsteinsson et al. 2009; Lowie et al. 2022).

### 4.4 Conservation Priorities

Our findings reinforce the role of tropical cities as overlooked reservoirs of vertebrate biodiversity. While our work presents the most comprehensive inventory of Guayaquil's herpetofauna to date, several gaps remain regarding the ecology, evolution, and behavior of these species, especially considering the impacts of urban pressure and pollution on their habitat (McDonald et al. 2019). The proximity of forests to the city has also led to an increase in wildlife‐human interactions, often resulting in several conflicts (Patterson et al. 2016). Although some citizens express a high degree of empathy toward wildlife, this is often diminished when the safety of pets or humans is negatively affected (Patterson et al. 2016). These wildlife‐human conflicts have also been a subject of analysis, with various entities, including government organizations, aiming to increase the visibility of urban fauna protection (Adams 2005; Collins et al. 2021). Addressing these conflicts through urban ecology research, education, and awareness campaigns will be crucial.

Moreover, conservation strategies must prioritize habitat preservation and restoration, increase monitoring of exotic and invasive species, and promote community engagement through educational initiatives to reduce wildlife‐human conflicts. Integrating climate change and land‐use change resilience into these efforts facilitates better adaptation of Guayaquil's herpetofauna diversity, making the city a model for balancing urban development and biodiversity conservation. Together, these results reinforce the role of tropical cities as overlooked reservoirs of vertebrate biodiversity and emphasize their potential contribution to conservation strategies in rapidly urbanizing regions.

## Author Contributions

**Keyko Cruz‐García:** conceptualization (equal), data curation (equal), formal analysis (equal), funding acquisition (equal), validation (equal), writing – original draft (equal), writing – review and editing (equal). **Natalia Zapata‐Salvatierra:** conceptualization (equal), data curation (equal), writing – original draft (supporting), writing – review and editing (supporting). **Andrea E. Narváez:** conceptualization (equal), data curation (equal), formal analysis (equal), funding acquisition (equal), resources (equal), validation (equal), writing – original draft (equal), writing – review and editing (equal). **Julián Pérez‐Correa:** conceptualization (equal), formal analysis (equal), funding acquisition (equal), resources (equal), writing – original draft (supporting), writing – review and editing (supporting).

## Funding

This work was supported by Universidad de Especialidades Espíritu Santo, Community dynamics of Guayas Province Herpetofauna.

## Conflicts of Interest

The authors declare no conflicts of interest.

## Supporting information


**Appendix S1:** Records obtained in this study. This includes the geographic coordinates, collection dates, sex, developmental stage and museum catalog numbers of the specimens examined, corresponding to the species recorded in the study area.

**Appendix S2:** List of herpetofauna species in Guayaquil obtained from own records, observer or collector information, specific localities within the city, historical museum codes, and bibliographic records. Sampling locations: Bosque y Vegetación Protectora Bosqueira (BOS); Bosque y Vegetación Protectora Cerro Blanco (CB); Bosque y Vegetación Protectora Cerro Paraíso (CPA); Bosque y Vegetación Protectora El Sendero Palo Santo (SPS); Bosque y Vegetación Protectora Prosperina (PRO); Bosque y Vegetación Protectora Cerro Colorado (CCO); Jardín Botánico de Guayaquil (GBG); Los Samanes National Recreation Area (SAM); Parque Lago National Recreation Area (PLA); El Salado Mangrove Wildlife Production Reserve (ESM); Parroquia Urbana Chongón, Guayaquil (CHO); Zoológico El Pantanal (PAN); Natural Science Faculty—Guayaquil University (NSF‐UG); Northern Urban Guayaquil (NUG); Southern Urban Guayaquil (SUG); Cerro Azul (AZU), Urban–Rural Generalist Habitat (URGH).

**Appendix S3:** Photographs of the individuals corresponding to the amphibian species recorded in the urban and peri‐urban areas of Guayaquil (Part 1). (1) *Rhinella bella*, (2) *Ceratophrys stolzmanni* , (3) *Craugastor longirostris* , (4) *Epipedobates machalilla* , (5) *Hyloxalus infraguttatus* , (6) *Boana rosenbergi*, (7) *Scinax quinquefasciatus* , (8) *Smilisca phaeota* . Photos: Keyko Cruz‐García and Natalia Zapata‐Salvatierra. (Part 2). (9) *Trachycephalus jordani* , (10) *Trachycephalus quadrangulum* , (11) *Engystomops pustulatus* , (12) *Engystomops randi* , (13) *Leptodactylus labrosus* , (14) *Leptodactylus melanonotus* . (15) *Leptodactylus ventrimaculatus* , (16) *Aquarana catesbeiana*. Photos: Keyko Cruz‐García and Natalia Zapata‐Salvatierra. Guayaquil (Part 3). (17) *Barycholos pulcher* , (18) *Pristimantis achatinus* , (19) *Caecilia tenuissima* . Photos: Keyko Cruz‐García and Natalia Zapata‐Salvatierra.

**Appendix S4:** Photographs of the individuals corresponding to the reptile species recorded in the urban and peri‐urban areas of Guayaquil (Part 1). (1) *Caiman crocodilus* , (2) *Crocodylus acutus* , (3) *Amphisbaena varia* , (4) *Alopoglossus festae*, (5) *Anolis binotatus* , (6) *Anolis festae* , (7) *Anolis gracilipes* , (8) *Anolis sagrei* . Photos: Keyko Cruz‐García and Natalia Zapata‐Salvatierra. (Part 2). (9) *Hemidactylus frenatus* , (10) *Iguana iguana* , (11) *Phyllodactylus reissii* , (12) *Polychrus femoralis* , (13) *Gonatodes caudiscutatus* , (14) *Lepidoblepharis buchwaldi* , (15) *Holcosus septemlineatus*, (16) *Medopheos edracanthus*. Photos: Keyko Cruz‐García and Natalia Zapata‐Salvatierra. (Part 3). (17) *Stenocercus iridescens* , (18) *Boa imperator* , (19) *Atractus microrhynchus* , (20) *Chironius flavopictus* , (21) *Clelia clelia* , (22) *Coniophanes dromiciformis* , (23) *Dendrophidion brunneum* , (24) *Dipsas georgejetti*. Photos: Keyko Cruz‐García and Natalia Zapata‐Salvatierra. (Part 4). (25) *Drymarchon melanurus* , (26) *Drymobius rhombifer* , (27) *Imantodes cenchoa* , (28) *Lampropeltis micropholis* , (29) *Leptodeira ornata*, (30) *Leptophis occidentalis*, (31) *Mastigodryas pulchriceps* , (32) *Mastigodryas reticulatus* . Photos: Keyko Cruz‐García and Natalia Zapata‐Salvatierra. (Part 5). (33) *Oxybelis transandinus*, (34) *Oxyrhopus petolarius* , (35) *Stenorrhina degenhardtii* , (36) *Tantilla capistrata* , (37) *Micrurus bocourti* , (38) *Micrurus mipartitus* , (39) *Epictia subcrotilla* , (40) *Indotyphlops braminus* . Photos: Keyko Cruz‐García, Melba Morán Soto and Natalia Zapata‐Salvatierra. (Part 6). (41) *Bothrops asper* , (42) *Chelydra acutirostris* , (43) *Rhinoclemmys annulata* , (44) *Kinosternon leucostomum* . Photos: Keyko Cruz‐García and Leonardo Alava.

## Data Availability

The data used in this manuscript are included as Appendixs S1–, S4. Further inquiries can be directed to the corresponding author.
