# Supplementary material for: Urban–Wild Interface Diversity: A Comprehensive Checklist of Herpetofauna of Guayaquil, Ecuador
Source: Ecol Evol. 2026 May 6;16(5):e73504. doi: 10.1002/ece3.73504 (PMC13149743; doi:10.1002/ece3.73504)
Supplement: Supplementary file 1 — Appendix S1: Records obtained in this study. This includes the geographic coordinates, collection dates, sex, developmental stage and museum catalog numbers of the specimens examined, corresponding to the species recorded in the study area. [file ECE3-16-e73504-s001.docx]

## Order Anura

### Family Bufonidae

***Rhinella bella* (Menéndez-Guerrero et al. 2024)**

**Material examined.** 1 (adult, ♂); 2° 10' 54.8" S, 80° 1' 5.1" W; alt. 37 m; 24.IV.2022; Keyko Cruz-García leg.; SVL 46 mm; ZSFQ4875 • 1 (adult, indet.); 2º 11' 57.96" S, 79º 51' 40.72" W; alt. 79 m; 17.III.2022; Jenny Barreno leg.; SVL 53.61 mm; QCAZA77745 • 1 (adult, ♂.); 2º 4' 46.71" S, 79º 54' 33.72" W; alt. 7 m; 30.III.2022; Jenny Barreno leg.; SVL 31.03 mm; QCAZA77749.

### Family Ceratophryidae

***Ceratophrys stolzmanni* (Steindachner 1882)**

**Material examined.** 1 (adult, ♂); 2° 8' 12.1" S, 80° 5' 23.5" W; alt. 387 m; 12.IV.2022; Keyko Cruz-García leg.; SVL 57 mm; ZSFQ4925 • 1 (adult, ♂); 2° 8' 52.4" S, 79° 58' 10.8" W; alt. 106 m; 14.III.2022; Jenny Barreno leg.; SVL 29.48 mm; QCAZA77754 • 1 (adult, indet.); 2º 9' 24.46" S, 79º 57' 59.33" W; alt. 230 m; 15.III.2022; Jenny Barreno leg.; SVL 29.48 mm; QCAZA77885.

### Family Craugastoridae

***Craugastor longirostris* (Boulenger 1898)**

**Material examined.** 1 (adult, indet.); 2° 10' 14.2" S, 80° 1' 50.71" W; alt. 275 m; 12.I.2016; Keyko Cruz-García, registration by observation.

### Family Dendrobatidae

***Epipedobates machalilla* (Coloma 1995)**

**Material examined.** 1 (adult, indet.); 2° 8' 32.5" S, 80° 5' 30.4" W; alt. 239 m; 12.IV.2022; Keyko Cruz-García leg.; SVL 15.94 mm; ZSFQ4892 • 1 (adult, indet.); 2° 7' 55.7" S, 80° 4' 57.5" W; alt. 198 m; 14.IV.2022; Natalia Zapata-Salvatierra leg.; SVL 13.03 mm; ZSFQ4894 • 1 (adult, indet.); 2° 8' 41.9" S, 79° 57' 40.4" W; alt. 87 m; 17.III.2022; Jenny Barreno leg.; SVL 15.73 mm; QCAZA77757.

***Hyloxalus infraguttatus* (Boulenger 1898)**

**Material examined.** 1 (adult, indet.); 2° 8' 44.4" S, 80° 5' 43..5" W; alt. 207 m; 12.IV.2022; Keyko Cruz-García leg.; SVL 19 mm; ZSFQ4879 • 1 (adult, ♀); 2° 7' 35.6" S, 80° 5' 1.5" W; alt. 190 m; 14.IV.2022; Keyko Cruz-García leg.; SVL 20.11 mm; ZSFQ4880 • 1 (Adult, indet.); 2º 9' 4.40" S, 79º 58' 41.04" W; alt. 251 m; 14.III.2022; Jenny Barreno leg.; SVL 15.8 mm; QCAZA77759.

### Family Hylidae

***Boana rosenbergi* (Boulenger 1898)**

**Material examined.** 1 (Adult, indet.); 2° 10' 11.9" S, 80° 1' 17.5" W; alt. 220 m; 16.VI.2016; Keyko Cruz-García, registration by observation.

***Scinax quinquefasciatus* (Fowler, 1913)**

**Material examined.** 1 (adult, indet.); 2º 9' 58.10" S, 80º 1' 39.47" W; alt. 245 m; 09.VII.2024; Keyko Cruz-García leg.; SVL 33.59 mm; MUTPL-SC-3674 • 1 (adult, indet.); 2° 9' 23.4" S, 79° 57' 47.2" W; alt. 169 m; 15.III.2022; Jenny Barreno leg.; SVL 33.8 mm; QCAZA77760.

***Smilisca phaeota* (Cope 1862)**

**Material examined.** 1 (Adult, indet.); 2° 10' 2.8" S, 79° 58' 59.8" W; alt. 102 m; 13.X.2021; Keyko Cruz-García, registration by observation • 1 (Adult, indet.); 2° 4' 48.2" S, 79° 54' 37.5" W; alt. 57 m; 29.VI.2022; Carlos Ruiz, registration by observation.

***Trachycephalus jordani* (Stejneger & Test 1891)**

**Material examined.** 1 (adult, indet.); 2° 10' 30.6" S, 80° 0' 59.8" W; alt. 245 m; 24.IV.2022; Keyko Cruz-García leg.; SVL 55 mm; ZSFQ4870 • 1 (adult, ♂); 2º 9' 8.08" S, 79º 57' 55.85" W; alt. 115 m; 17.III.2022; José de los Ángeles leg.; SVL 88.3 mm; QCAZA77769 • 1 (adult, indet.); 2º 6' 34.21" S, 79º 55' 27.05" W; alt. 4 m; 10.V.2022; Stefania Cuadrado leg.; SVL 52.34 mm; QCAZA77937.

***Trachycephalus quadrangulum* (Boulenger 1882)**

**Material examined.** 1 (adult, indet.); 2° 8' 39" S, 80° 5' 5.9" W; alt. 234 m; 10.IV.2022; Keyko Cruz-García leg.; SVL 60.32 mm; ZSFQ4872 • 1 (adult, indet.); 2° 10' 4.5" S, 79° 58' 53.5" W; alt. 105 m; 11.V.2022; Keyko Cruz-García leg.; SVL 70.01 mm; ZSFQ4873 • 1 (adult, indet.); 2° 8' 14.1" S, 80° 5' 19.1" W; alt. 377 m; 21.VII.2022; Keyko Cruz-García leg.; SVL 65.20 mm; QCAZA78034.

### Family Leptodactylidae

***Engystomops pustulatus* (Ron et al. 2005)**

**Material examined.** 1 (adult, indet.); 2° 8' 29.5" S, 80° 4' 17.6" W; alt. 361 m; 12.IV.2022; Keyko Cruz-García leg.; SVL 28.01 mm; ZSFQ4888 • 1 (adult, ♂); 2º 10' 15.06" S, 79º 55' 31.69" W; alt. 91 m; 22.III.2022; Jenny Barreno leg.; SVL 25.2 mm; QCAZA77895 • 1 (adult, ♀); 2º 9' 31,66" S, 79º 54' 7.82" W; alt. 27 m; 30.III.2022; Jenny Barreno leg.; SVL 25.9 mm; QCAZA77813.

***Engystomops randi* (Ron et al. 2004)**

**Material examined.** 1 (adult, indet.); 2° 9' 58.7" S, 80° 0' 25.7" W; alt. 349 m; 08.V.2022; Keyko Cruz-García leg.; SVL 25 mm; ZSFQ4890 • 1 (adult, indet.); 2° 9' 15.3" S, 79° 59' 18.1" W; alt. 401 m; 08.V.2022; Keyko Cruz-García leg.; SVL 23 mm; ZSFQ4891.

***Leptodactylus labrosus* (Jiménez de la Espada 1875)**

**Material examined.** 1 (adult, indet.); 2° 8' 9.3" S, 80° 5' 26.4" W; alt. 380 m; 10.IV.2022; Keyko Cruz-García leg.; SVL 30.3 mm; ZSFQ4876 • 1 (adult, indet.); 2º 10' 17.71" S, 79º 55' 37.94" W; alt. 181 m; 22.III.2022; Jenny Barreno leg.; SVL 23.4 mm; QCAZA77900 • 1 (adult, ♂); 2º 6' 25.22" S, 79º 55' 16.80" W; alt. 8 m; 11.V.2022; Stefania Cuadrado leg.; SVL 39.98 mm; QCAZA77960.

***Leptodactylus melanonotus* (Hallowell 1861)**

**Material examined.** 1 (adult, indet.); 2º 8' 16.84" S, 80º 4' 50.82" W; alt. 367 m; 12.IV.2022; Natalia Zapata-Salvatierra, registration by observation • 1 (adult, indet.); 2º 9' 43.43" S, 80º 3' 24.64" W; alt. 380 m; 13.IV.2022; Keyko Cruz-García, registration by observation • 1 (adult, indet.); 2º 9' 28.84" S, 80º 2' 28.81" W; alt. 154 m; 22.V.2022; Natalia Zapata-Salvatierra, registration by observation.

***Leptodactylus ventrimaculatus*** **(Boulenger 1902)**

**Material examined.** 1 (adult, indet.); 2° 9' 23.2" S, 79° 57' 47.2" W; alt. 182 m; 15.III.2022; Jenny Barreno leg.; SVL 31.23 mm; QCAZA77843 • 1 (adult, indet.); 2º 9' 23.22" S, 79º 57' 47.16" W; alt. 119 m; 15.III.2022; Jenny Barreno leg.; SVL 20.49 mm; QCAZA77847.

***Aquarana catesbeiana* (Dubois et al. 2021)**

**Material examined.** 1 (juvenil, indet); 2º 9' 26.3" S, 79º 57' 55.9" W; alt. 216 m; 18.IV.2022; Stefania Cuadrado, QCAZA77847 •1 (adult, indet); 2º 8' 50.70" S, 80º 6' 44.70" W; alt. 133 m; 27.VII.2024; Keyko Cruz-García, registration by observation • 1 (adult, indet); 2º 8' 51.25" S, 80º 6' 44.23" W; alt. 133 m; 02.VIII.2024; Keyko Cruz-García, registration by observation.

### Family Strabomantidae

***Barycholos pulcher* (Boulenger 1898)**

**Material examined.** 1 (adult, indet.); 2° 10' 11.9" S, 80° 2' 28.3" W; alt. 276 m; 13.II.2015; Keyko Cruz-García, registration by observation • 1 (adult, indet.); 2° 8' 50.1" S, 79° 55' 5.7" W; alt. 20 m; 27.VII.2014; Keyko Cruz-García, registration by observation.

***Pristimantis achatinus* (Boulenger 1898)**

**Material examined.** 1 (adult, indet.); 2° 10' 13" S, 80° 1' 35" W; alt. 210 m; 25.IV.2022; Keyko Cruz-García leg.; SVL 35 mm; ZSFQ4884 • 1 (adult, indet.); 2° 9' 45.6" S, 79° 59' 25.6" W; alt. 256 m; 08.V.2022; Keyko Cruz-García leg.; SVL 33 mm; ZSFQ4887 • 1 (adult, ♂); 2º 9' 1.63" S, 79º 58' 41.88" W; alt. 239 m; 14.III.2022; Jenny Barreno leg.; SVL 32.9 mm; QCAZA77849.

## Order Gymnophiona

### Family Caeciliidae

***Caecilia tenuissima* (Taylor 1973)**

**Material examined.** 1 (adult, ♂); 2° 8' 11.9" S, 80° 4' 37" W; alt. 391 m; 12.IV.2022; Keyko Cruz-García leg.; SVL 623 mm; ZSFQ4898.

Class Reptilia

## Order Crocodylia

### Family Alligatoridae

***Caiman crocodilus* (Linnaeus 1758)**

**Material examined.** 1 (juvenil, indet.); 2º 12' 58.96" S, 80º 5' 39.89" W; alt. 47 m; 26.VII.2024; Keyko Cruz-García, registration by observation • 1 (juvenil, indet.); 2º 12' 56.85" S, 80º 5' 37.72" W; alt. 44 m; 26.VII.2024; Keyko Cruz-García, registration by observation • 1 (juvenil, indet.); 2º 12' 16.44" S, 80º 9' 51.51" W; alt. 55 m; 18.VIII.2024; Jaime Arellano, registration by observation.

### Family Crocodylidae

***Crocodylus acutus* (Cuvier 1807)**

**Material examined.** 1 (adult, indet.); 2º 11' 45.39" S, 79º 58' 10.40" W; alt. 11 m; 11.I.2018; Keyko Cruz-García, registration by observation • 1 (adult, indet.); 2º 11' 35.51" S, 80º 1' 25.12" W; alt. 10 m; 21.VI.2016; Keyko Cruz-García, registration by observation • 1 (adult, indet.); 2º 11' 29.34" S, 79º 59' 15.19" W; alt. 9 m; 10.IX.2023; Benjamin Navas, registration by observation.

## Order Squamata: Amphisbaenia

### Family Amphisbaenidae

***Amphisbaena fuliginosa varia* (Laurenti 1768)**

**Material examined.** 1 (adult, indet.); 2º 9' 56.23" S, 80º 2' 19.88" W; alt. 292 m; 18. VIII.2017; Keyko Cruz-García, registration by observation • 1 (adult, indet.); 2º 10' 7.74" S, 80º 0' 45.43" W; alt. 283 m; 02.II.2015; Keyko Cruz-García, registration by observation.

## Order Squamata: Sauria

### Family Anolidae

***Anolis binotatus* (Peters 1863a)**

**Material examined.** 1 (adult, indet.); 2º 8' 7.90" S, 80º 5' 9.60" W; alt. 265 m; 21.VII.2022; Keyko Cruz-García leg.; SVL 54 mm; QCAZR17985 • 1 (adult, indet.); 2º 9' 42.34" S, 79º 59' 36.17" W; alt. 381 m; 11.X.2021; Keyko Cruz-García, registration by observation.

***Anolis festae* (Peracca 1904)**

**Material examined.** 1 (adult, ♂); 2º 9' 59.78" S, 80º 1' 11.59" W; alt. 305 m; 27.IV.2022; Keyko Cruz-García leg.; SVL 47 mm; ZSFQ4918 • 1 (adult, indet.); 2º 9' 28.62" S, 79º 59' 43.18" W; alt. 354 m; 02.VIII.2024; Keyko Cruz-García leg.; SVL 51.96 mm; MUTPL-R 598.

***Anolis gracilipes* (Boulenger 1898)**

**Material examined.** 1 (adult, ♀); 2º 8' 14.14" S, 80º 5' 19.09" W; alt. 371 m; 12.IV.2022; Natalia Zapata-Salvatierra leg.; SVL 55 mm; ZSFQ4917 • 1 (adult, indet.); 2º 9' 46.53" S, 79º 59' 21.60" W; alt. 254 m; 11.IX.2021; Keyko Cruz-García, registration by observation.

***Anolis sagrei* (Duméril & Bibron 1837)**

**Material examined.** 1 (adult, ♀); 2º 9' 54.97" S, 79º 53' 56.73" W; alt. 14 m; 30.III.2022; Jenny Barreno leg.; SVL 33.7 mm; QCAZR17891 • 1 (adult, indet.); 2º 0' 26.95" S, 79º 58' 45.77" W; alt. 63 m; 23.VIII.2022; Stefania Cuadrado leg.; SVL 68.49 mm; QCAZR17989.

### Family Alopoglossidae

***Alopoglossus harrisi* (Hernández et al. 2020)**

**Material examined.** 1 (juvenil, indet.); 2º 10' 24.6" S, 80º 1' 19.07" W; alt. 142 m; 26.IV.2022; Natalia Zapata-Salvatierra leg.; SVL 50 mm; ZSFQ4916 • 1 (adult, ♂); 2º 10' 6.64" S, 80º 0' 38.99" W; alt. 318 m; 09.VII.2024; Keyko Cruz-García leg.; SVL 50.10 mm; MUTPL-R 596.

### Family Gekkonidae

***Hemidactylus frenatus* (Duméril & Bibron 1836)**

**Material examined.** 1 (adult, indet.); 2º 10' 55.02" S, 80º 1' 5.05" W; alt. 38 m; 24.IV.2022; Keyko Cruz-García leg.; SVL 39 mm; ZSFQ4911 • 1 (adult, ♂); 2º 9' 1.92" S, 79º 57' 40.09" W; alt. 83 m; 17.III.2022; Jenny Barreno leg.; SVL 31.05 mm; QCAZR17878.

### Family Iguanidae

***Iguana iguana* (Linnaeus 1758)**

**Material examined.** 1 (adult, ♂); 2º 10' 9.45" S, 80º 1' 34.69" W; alt. 205 m; 29.IX.2021; Natalia Zapata-Salvatierra, registration by observation • 1 (adult, indet.); 2º 6' 14.03"S, 79º 54' 48.75" W; alt. 11 m; 16.IV.2017; Keyko Cruz-García, registration by observation.

### Family Phyllodactylidae

***Phyllodactylus reissii* (Peters 1862)**

**Material examined.** 1 (adult, indet.); 2º 10' 49.16" S, 80º 1' 2.29" W; alt. 81 m; 24.IV.2022; Keyko Cruz-García leg.; SVL 67 mm; ZSFQ4910 • 1 (adult, ♂); 2º 8' 25.19" S, 80º 5' 18.86" W; alt. 254 m; 19.VII.2022; Keyko Cruz-García leg.; SVL 62 mm; QCAZR18001.

### Family Polychrotidae

***Polychrus femoralis* (Werner 1910)**

**Material examined.** 1 (adult, indet.); 2º 9' 7.72" S, 80º 4' 44.40" W; alt. 56 m; 13.II.2016; Keyko Cruz-García, registration by observation • 1 (adult, indet.); 2º 8' 34.14" S, 80º 2' 39.14" W; alt. 168 m; 12.X.2021; Natalia Zapata-Salvatierra, registration by observation • 1 (adult, indet.); 2º 10' 42.34" S, 80º 0' 58.93" W; alt. 161 m; 21.IV.2024; Kevin Peñafiel, registration by observation (Fig. 3.12).

### Family Spheaerodactylidae

***Gonatodes caudiscutatus* (Günther 1859)**

**Material examined.** 1 (adult, indet.); 2º 8' 9.81" S, 80º 4' 50.3" W; alt. 299 m; 14.IV.2022; Natalia Zapata-Salvatierra leg.; SVL 39 mm; ZSFQ4912 • 1 (adult, ♀); 2º 10' 30.14" S, 80º 1' 9.33" W; alt. 175 m; 25.IV.2022; Keyko Cruz-García leg.; SVL 37 mm; ZSFQ4914 • 1 (adult, ♂); 2º 10' 21.25" S, 80º 1' 15.70" W; alt. 153 m; 25.IV.2022; Natalia Zapata-Salvatierra leg.; SVL 34 mm; ZSFQ4915.

***Lepidoblepharis buchwaldi* (Werner 1910)**

**Material examined.** 1 (adult, indet.); 2º 8' 13.68" S, 80º 4' 48.29" W; alt. 368 m; 14.IV.2022; Keyko Cruz-García leg.; SVL 18 mm; ZSFQ4913 • 1 (juvenil, **♂**); 2º 8' 45.87" S, 79º 57' 45.05" W; alt. 81 m; 17.III.2022; Jenny Barreno leg.; SVL 10.78 mm; QCAZR17928.

### Family Teiidae

***Holcosus septemlineatus* (Duméril & Duméril 1851)**

**Material examined.** 1 (juvenil, ♀); 2º 8' 42.53" S, 80º 5' 10.48" W; alt. 226 m; 10.IV.2022; Natalia Zapata-Salvatierra leg.; SVL 70.3 mm; ZSFQ4923 • 1 (juvenil, indet.); 2º 8' 17.77" S, 80º 5' 16.54" W; alt. 370 m; 12.VII.2022; Keyko Cruz-García.; QCAZR18022.

***Medopheos edracanthus* (Bocourt 1874)**

**Material examined.** 1 (adult, ♂); 2º 10' 53.36" S, 80º 1' 3.10" W; alt. 53 m; 26.IV.2022; Keyko Cruz-García leg.; SVL 69 mm; ZSFQ4924 • 1 (adult, indet.); 2º 10' 1.41" S, 80º 1' 40.85" W; alt. 241 m; 24.IV.2022; Keyko Cruz-García, registration by observation • 1 (adult, indet.); 2º 10' 4.29" S, 80º 1' 9.61" W; alt. 309 m; 26.X.2021; Natalia Zapata-Salvatierra, registration by observation.

### Family Tropiduridae

***Stenocercus iridescens* (Günther 1859)**

**Material examined.** 1 (adult, ♀); 2º 10' 31.08" S, 80º 1' 8.90" W; alt. 209 m; 26.IV.2022; Keyko Cruz-García leg.; SVL 71 mm; ZSFQ4921 • 1 (adult, ♂); 2º 8' 34.50" S, 80º 5' 13.56" W; alt. 300 m; 11.IV.2022; Natalia Zapata-Salvatierra leg.; SVL 93 mm; ZSFQ4922 • 1 (adult, ♂); 2º 6' 20.11" S, 79º 54' 34.63" W; alt. 8 m; 09.V.2022; Stefania Cuadrado leg.; SVL 78 mm; QCAZR17941.

## Order Squamata: Serpentes

### Family Boidae

***Boa imperator* (Daudin 1803a)**

**Material examined.** 1 (adult, indet.); 2º 10' 29.66" S, 80º 1' 17.20" W; alt. 129 m; 26.IV.2022; Keyko Cruz-García, registration by observation • 1 (adult, indet.); 2º 4' 20.18" S, 79º 54' 57.90" W; alt. 39 m; 23.V.2013; Keyko Cruz-García, registration by observation • 1 (adult, indet.); 2° 8' 46.30" S, 79° 58' 28.52" W; alt. 145 m; 20.VII.2023; Julian Perez-Correa, registration by observation.

### Family Colubridae

***Atractus microrhynchus* (Cope 1868)**

**Material examined.** 1 (adult, indet.); 2º 8' 37.25" S, 80º 5' 33.29" W; alt. 242 m; 10.IV.2022; Keyko Cruz-García leg.; SVL 393 mm; ZSFQ4899 • 1 (adult, ♀); 2º 9' 19.51" S, 79º 59' 27.36" W; alt. 402 m; 09.V.2022; Keyko Cruz-García leg.; SVL 265 mm; ZSFQ4919 • 1 (adult, ♂); 2º 9' 28.11" S, 79º 59' 37.42" W; alt. 399 m; 09.V.2022; Natalia Zapata-Salvatierra.; SVL 275 mm; ZSFQ4920.

***Chironius flavopictus* (Werner 1909)**

**Material examined.** 1 (adult, indet.); 2º 10' 32.74" S, 80º 1' 5.68" W; alt. 225 m; 20.III.2015; Keyko Cruz-García, registration by observation • 1 (adult, indet.); 2º 9' 30.62" S, 79º 58' 47.81" W; alt. 326 m; 05.XII.2023; Juan de Dios Morales, registration by observation.

***Clelia clelia* (Daudin 1803a)**

**Material examined.** 1 (adult, indet.); 2º 8' 40.47" S, 80º 4' 53.05" W; alt. 262 m; 10.IV.2022; Keyko Cruz-García, registration by observation.

***Coniophanes dromiciformis* (Peters 1863b)**

**Material examined.** 1 (adult, ♂); 2º 10' 26.70" S, 80º 1' 16.91" W; alt. 132 m; 24.IV.2022; Keyko Cruz-García leg.; SVL 351 mm; ZSFQ4904 • 1 (adult, ♂); 2º 9' 14.35" S, 79º 58' 41.73" W; alt. 383 m; 12.V.2022; Keyko Cruz-García leg.; SVL 244 mm; ZSFQ4905 • 1 (adult, indet.); 2º 9' 31.60" S, 80º 3' 29.74" W; alt. 306 m; 06.VIII.2024; Keyko Cruz-García leg.; SVL 290.50 mm; MUTPL-R 605.

***Dendrophidion brunneum* (Günther 1858)**

**Material examined.** 1 (adult, indet.); 2º 10' 23.72" S, 80º 1' 24.99" W; alt. 232 m; 09.VII.2024; Keyko Cruz-García leg.; SVL 250.30 mm; MUTPL-R 595 • 1 (adult, indet.); 2º 10' 19.16" S, 80º 1' 0.73" W; alt. 316 m; 13.V.2015; Keyko Cruz-García, registration by observation • 1 (adult, indet.); 2º 8' 16.84" S, 80º 4' 50.82" W; alt. 367 m; 12.IV.2022; Natalia Zapata-Salvatierra, registration by observation.

***Dipsas georgejetti* (Arteaga et al. 2018)**

**Material examined.** 1 (adult, ♀); 2º 8' 42.48" S, 79º 57' 47.71" W; alt. 79 m; 17.III.2022; Jenny Barreno leg.; SVL 280.5 mm; QCAZR17871 • 1 (adult, indet.); 2º 0' 39.35" S, 79º 58' 33.63" W; alt. 66 m; 19.IV.2022; Stefania Cuadrado leg.; SVL 300.5 mm; QCAZR17872.

***Drymarchon melanurus* (Duméril et al. 1854)**

**Material examined.** 1 (adult, indet.); 2º 6' 23.52" S, 79º 5' 52.0" W; alt. 18 m; 09.VIII.2018; Keyko Cruz-García, registration by observation • 1 (adult, indet.); 2º 9' 10.58" S, 79º 5' 13.1" W; alt. 430 m; 11.IX.2021; Keyko Cruz-García, registration by observation • 1 (adult, indet.); 2º 8' 22.63" S, 80º 5' 28.21" W; alt. 313 m; 12.IV.2022; Natalia Zapata-Salvatierra, registration by observation.

***Drymobius rhombifer* (Günther 1860)**

**Material examined.** 1 (adult, indet.); 2º 8' 9.96" S, 80º 4' 6.66" W; alt. 279 m; 22.II.2025; Keyko Cruz-García leg.; SVL 52.80 mm; MUTPL-R 646 • 1 (adult, indet.); 2º 10' 21.01" S, 80º 1' 22.65" W; alt. 193 m; 22.VI.2024; Mauricio Andrés Romero, registration by observation.

***Imantodes cenchoa* (Linnaeus 1758)**

**Material examined.** 1 (adult, indet.); 2º 8' 6.42" S, 80º 4' 58.84" W; alt. 282 m; 04.VIII.2024; Keyko Cruz-García leg.; SVL 340.30 mm; MUTPL-R 601 • 1 (adult, indet.); 2º 10' 4.29" S, 80º 1' 9.61" W; alt. 309 m; 26.X.2021; Natalia Zapata-Salvatierra, registration by observation.

***Lampropeltis micropholis* (Cope 1860)**

**Material examined.** 1 (adult, indet.); 2º 10' 27.11" S, 80º 1' 17.81" W; alt. 146 m; 01.III.2015; Keyko Cruz-García, registration by observation • 1 (adult, indet.); 2º 12' 36.83" S, 80º 5' 3.64" W; alt. 55 m; 28.XI.2023; Juan de Dios Morales, registration by observation.

***Leptodeira ornata* (Bocourt 1884)**

**Material examined.** 1 (adult, indet.); 2º 10' 4.98"S, 80º 1' 40.49" W; alt. 212 m; 25.IV.2022; Keyko Cruz-García leg.; SVL 578 mm; ZSFQ4900 • 1 (adult, indet.); 2º 7' 55.48" S, 80º 3' 56.21" W; alt. 277 m; 06.VIII.2024; Keyko Cruz-García leg.; SVL 700.90 mm; MUTPL-R 606.

***Leptophis occidentalis* (Günther 1859)**

**Material examined.** 1 (adult, indet.); 2º 9' 3.96" S, 80º 4' 18.12" W; alt. 277 m; 04.VIII.2024; Keyko Cruz-García leg.; SVL 460.10 mm; MUTPL-R 603 • 1 (adult, indet.); 2º 9' 44.51" S, 79º 58' 29.00" W; alt. 139 m; 25.V.2022; Keyko Cruz-García, registration by observation • 1 (adult, indet.); 2º 8' 46.30" S, 79º 55' 2.49" W; alt. 22 m; 16.VI.2015; Keyko Cruz-García, registration by observation.

***Mastigodryas pulchriceps* (Cope 1868)**

**Material examined.** 1 (adult, indet.); 2º 9' 17.88" S, 80º 3' 32.82" W; alt. 309 m; 07.I.2021; Keyko Cruz-García, registration by observation.

***Mastigodryas reticulatus* (Peters 1863b)**

**Material examined.** 1 (adult, ♀); 2º 10' 31.47" S, 80º 0' 58.96" W; alt. 234 m; 24.IV.2022; Keyko Cruz-García leg.; SVL 735 mm; ZSFQ4901 • 1 (adult, ♂); 2º 10' 36.11" S, 80º 1' 15.04" W; alt. 127 m; 10.XII.2023; Keyko Cruz-García leg.; SVL 800 mm; MUTPL-R 472.

***Oxybelis transandinus* (Torres-Carvajal et al. 2021)**

**Material examined.** 1 (adult, ♀); 2º 9' 18.56" S, 79º 59' 25.52" W; alt. 401 m; 09.V.2022; Keyko Cruz-García leg.; SVL 677 mm; ZSFQ4902 • 1 (adult, indet.); 2º 9' 47.50" S, 79º 59' 46.37" W; alt. 297 m; 02.VIII.2024; Keyko Cruz-García leg.; SVL 850.50 mm; MUTPL-R 599.

***Oxyrhopus petolarius* (Linnaeus 1758)**

**Material examined.** 1 (adult, indet.); 2º 8' 29.27" S, 80º 5' 8.51" W; alt. 274 m; 24.V.2024; Keyko Cruz-García leg.; SVL 400 mm; MUTPL-R 593 • 1 (adult, indet.); 2º 8' 9.28" S, 80º 5' 25.08" W; alt. 380 m; 10.VII.2024; Keyko Cruz-García leg.; SVL 290.20 mm; MUTPL-R 594 • 1 (adult, indet.); 2º 8' 54.87" S, 80º 5' 1.08" W; alt. 328 m; 04.VIII.2024; Keyko Cruz-García leg.; SVL 430.60 mm; MUTPL-R 602.

***Stenorrhina degenhardtii* (Berthold 1846)**

**Material examined.** 1 (adult, indet.); 2º 8' 10.50" S, 80º 5' 3.00" W; alt. 366 m; 25.V.2022; Keyko Cruz-García leg.; SVL 362 mm; ZSFQ4903.

***Tantilla capistrata* (Cope 1875)**

**Material examined.** 1 (adult, ♂); 2° 9' 19.24" S, 79° 57' 32.63" W; alt. 118 m; 16.I.2024; Michelle Vella Torres leg.; SVL 265 mm; MUTPL-R 490 • 1 (adult, indet.); 2º 9' 21.63" S, 80º 2' 8.64" W; alt. 315 m; 11.VII.2024; Keyko Cruz-García leg.; SVL 210.80 mm; MUTPL-R 597.

### Family Elapidae

***Micrurus bocourti* (Jan 1872)**

**Material examined.** 1 (adult, indet.); 2º 8' 9.53" S, 80º 5' 26.79" W; alt. 367 m; 10.IV.2022; Keyko Cruz-García leg.; SVL 703 mm; ZSFQ4908 • 1 (adult, indet.); 2º 10' 17.85" S, 80º 1' 29.88" W; alt. 185 m; 25.IV.2022; Natalia Zapata-Salvatierra leg.; SVL 769 mm; ZSFQ4909 • 1 (adult, indet.); 2º 9' 2.3" S, 80º 2' 55.06" W; alt. 300 m; 06.VIII.2024; Keyko Cruz-García leg.; SVL 550.90 mm; MUTPL-R 604.

***Micrurus mipartitus* (Duméril et al. 1836)**

**Material examined.** 1 (adult, indet.); 2º 10' 21.24" S, 79º 57' 2.78" W; alt. 313 m; 04.V.2024; Melba Morán Soto, registration by observation.

### Family Leptotyphlopidae

***Epictia subcrotilla* (Klauber 1939)**

**Material examined.** 1 (adult, indet.); 2º 7' 49.04" S, 79º 53' 35.03" W; alt. 14 m; 29.XII.2023; Keyko Cruz-García leg.; SVL 86 mm; MUTPL-R 489 • 1 (adult, indet.); 2º 10' 40.18" S, 80º 1' 5.87" W; alt. 169 m; 14.V.2015; Keyko Cruz-García, registration by observation • 1 (adult, indet.); 2º 5' 58.88" S, 79º 54' 49.01" W; alt. 8 m; 19.VII.2018; Keyko Cruz-García, registration by observation.

### Family Typhlopidae

***Indotyphlops braminus*** **(Daudin 1803b)**

**Material examined.** 1 (adult, indet.); 2º 10' 30.53" S, 79º 53' 56.42" W, alt. 5.1 m; 15.III.2024; Leonardo Alava leg.; SVL 164 mm; MUTPL-R 653.

### Family Viperidae

***Bothrops asper* (Garman 1884)**

**Material examined.** 1 (juvenil, indet.); 2º 8' 38.13" S, 80º 5' 1.07" W; alt. 234 m; 10.IV.2022; Keyko Cruz-García leg.; SVL 398 mm; ZSFQ4906 • 1 (juvenil, indet.); 2º 9' 55.54" S, 79º 58' 57.51" W; alt. 154 m; 08.V.2022; Keyko Cruz-García leg.; SVL 256 mm; ZSFQ4907.

## Order Testudines

### Family Chelydridae

***Chelydra acutirostris* (****Peters 1862)**

**Material examined.** 1 (adult, indet.); 2º 6' 14.03" S, 79º 54' 48.75" W; alt. 11 m; 24.XI.2019; Keyko Cruz-García, registration by observation • 1 (adult, indet.); 2º 13' 38.76" S, 79º 55' 35.35" W; alt. 3 m; 16.VII.2022; Kevin Peñafiel, registration by observation • 1 (adult, indet.); 2° 8' 40.97" S, 79° 58' 1.75" W; alt. 80 m; 16.II.2024; Michelle Vela Torres, registration by observation.

### Family Geoemydidae

***Rhinoclemmys annulata* (Gray 1860)**

**Material examined.** 1 (adult, indet.); 2º 8' 16.84" S, 80º 4' 50.82" W; alt. 367 m; 15.X.2022; Keyko Cruz-García, registration by observation • 1 (adult, indet.); 2° 8' 36.09" S, 79° 58' 1.03" W; alt. 80 m; 03.IV.2024; Michelle Vela Torres, registration by observation • 1 (adult, indet.); 2º 10' 52.90" S, 80º 1' 10.99" W; alt. 42 m; 18.IV.2023; Daniel López, registration by observation.

### Family Kinosternidae

***Kinosternon leucostomum* (Duméril & Duméril 1851)**

**Material examined.** 1 (adult, indet.); 2º 10' 39.82" S, 80º 1' 6.23" W; alt. 170 m; 28.VII.2017; Keyko Cruz-García, registration by observation.
