# Supplementary figures and images for: Urban–Wild Interface Diversity: A Comprehensive Checklist of Herpetofauna of Guayaquil, Ecuador
Source: Ecol Evol. 2026 May 6;16(5):e73504. doi: 10.1002/ece3.73504 (PMC13149743; doi:10.1002/ece3.73504)

1

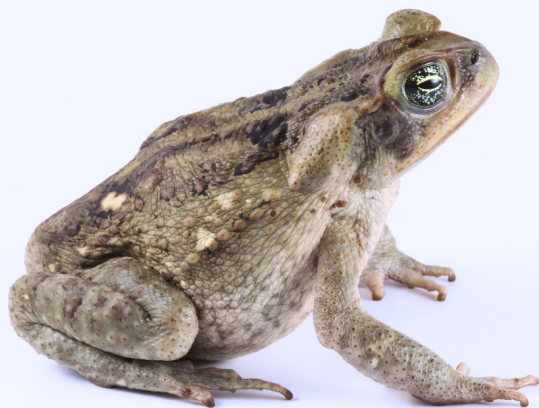

2

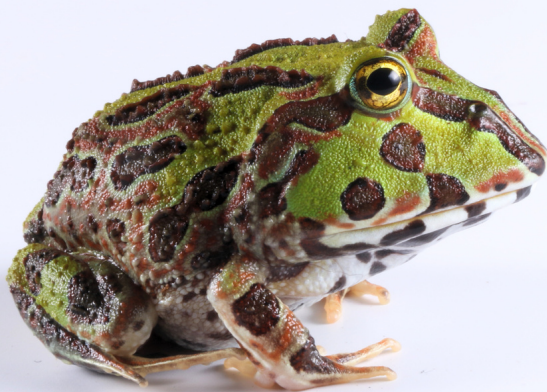

3

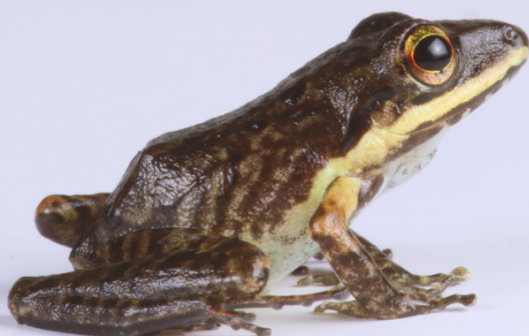

4

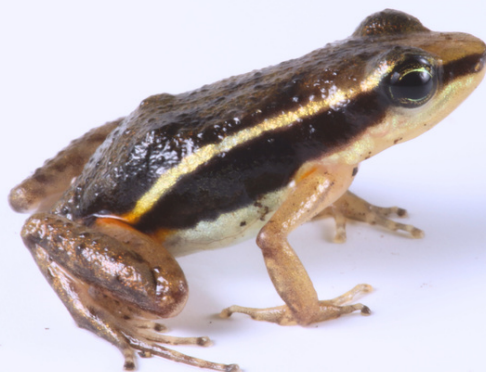

5

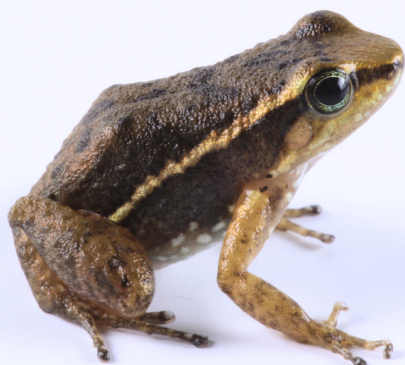

6

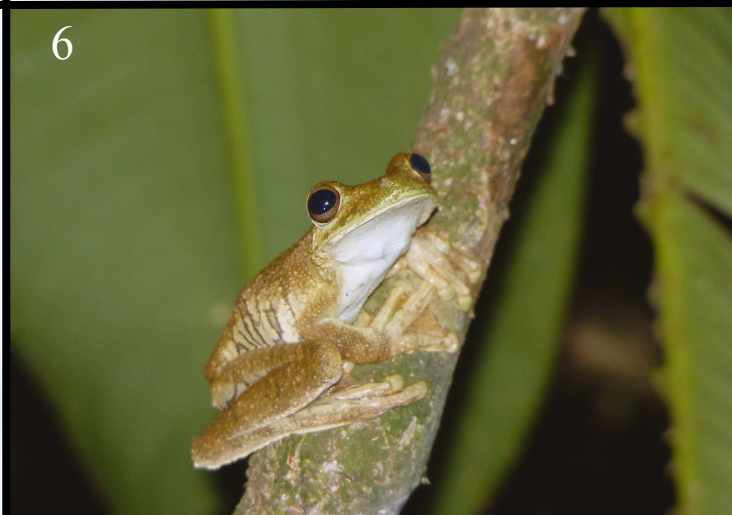

7

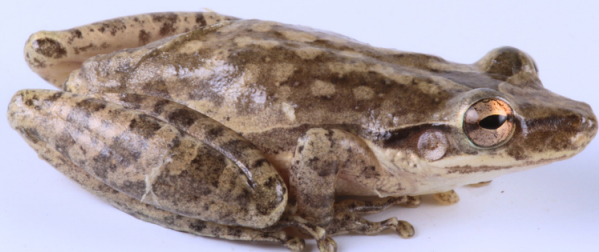

8

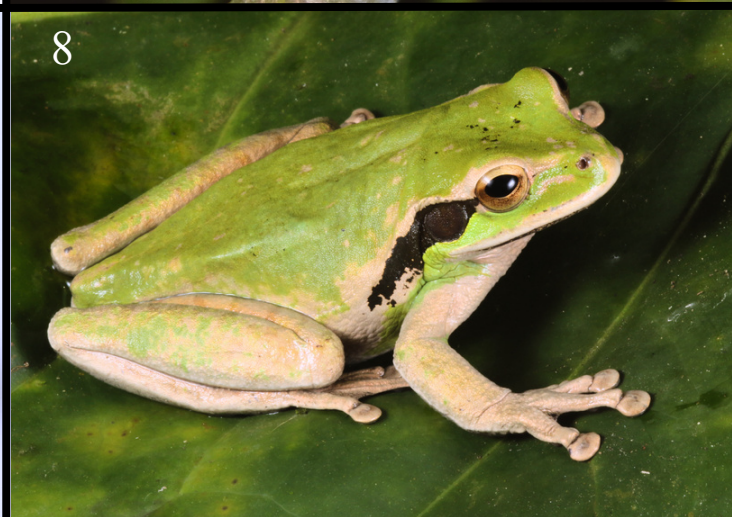

9

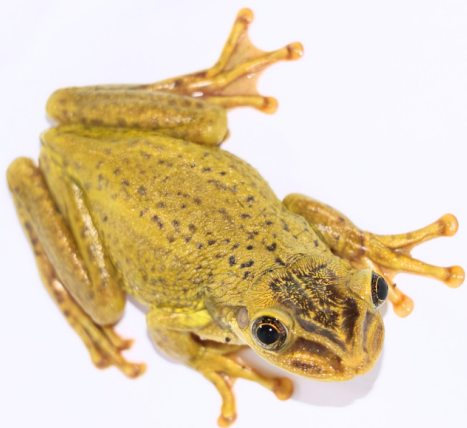

10

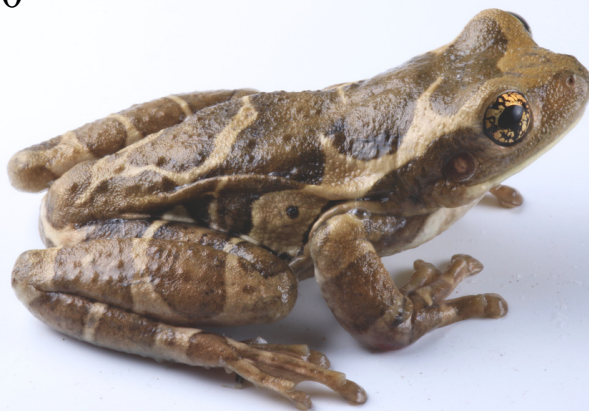

11

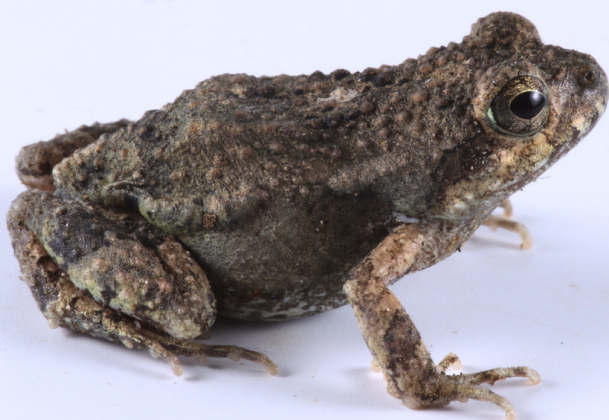

12

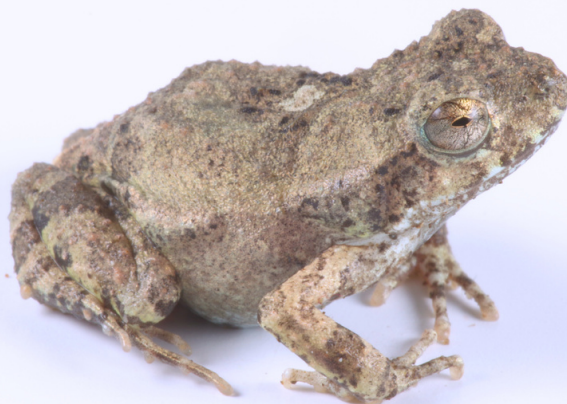

13

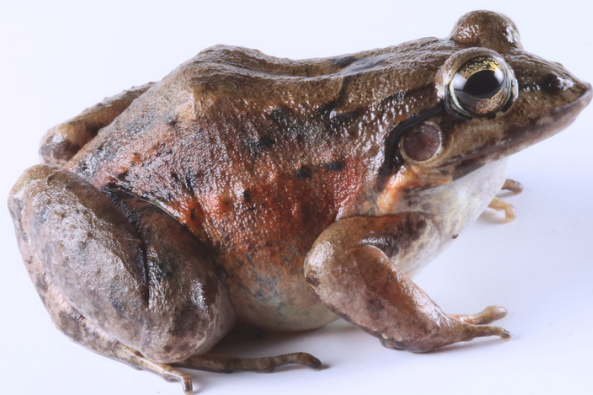

14

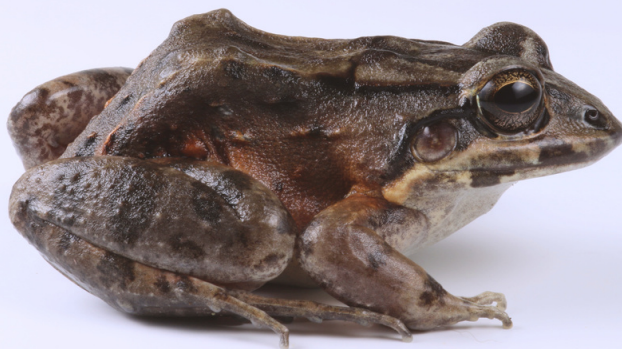

15

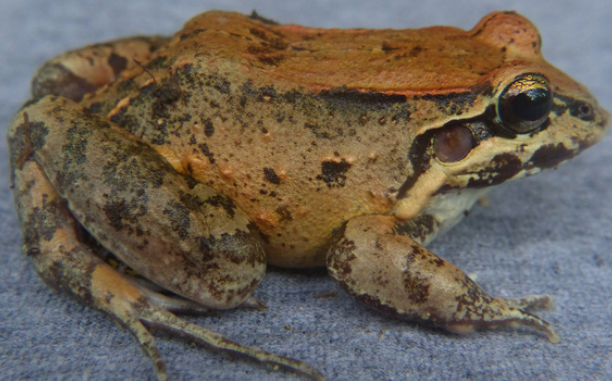

16

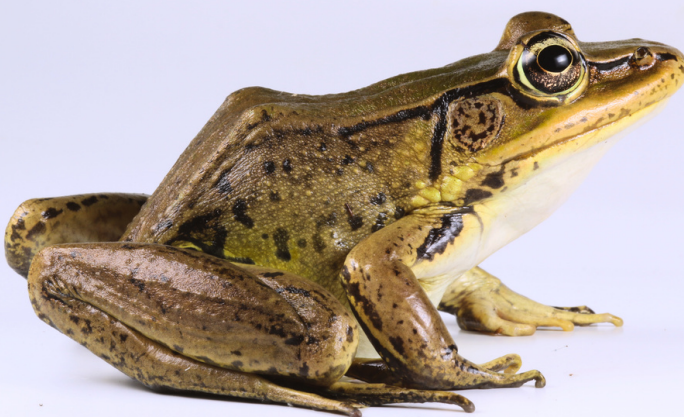

17

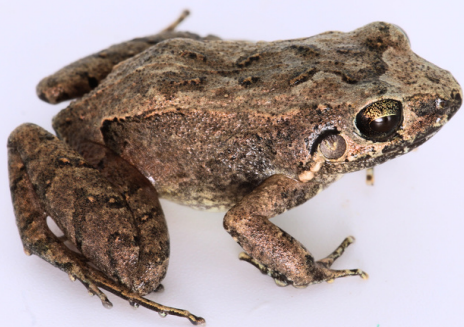

18

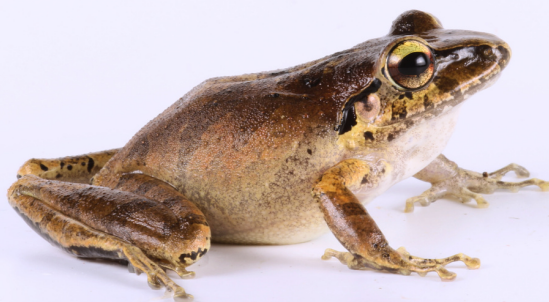

19

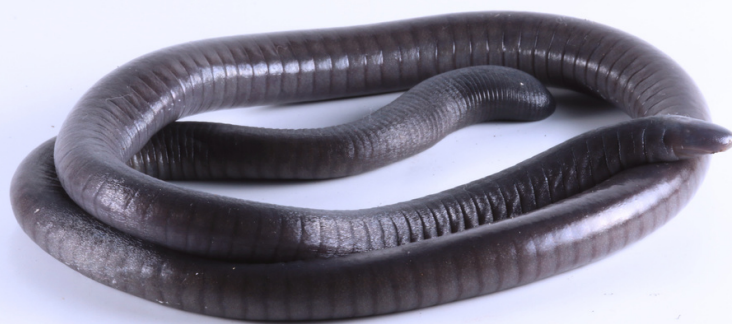

Supplement: Supplementary file 3 — Appendix S3: Photographs of the individuals corresponding to the amphibian species recorded in the urban and peri‐urban areas of Guayaquil (Part 1). (1) Rhinella bella, (2) Ceratophrys stolzmanni , (3) Craugastor longirostris , (4) Epipedobates machalilla , (5) Hyloxalus infraguttatus , (6) Boana rosenbergi, (7) Scinax quinquefasciatus , (8) Smilisca phaeota . Photos: Keyko Cruz‐García and Natalia Zapata‐Salvatierra. (Part 2). (9) Trachycephalus jordani , (10) Trachycephalus quadrangulum , (11) Engystomops pustulatus , (12) Engystomops randi , (13) Leptodactylus labrosus , (14) Leptodactylus melanonotus . (15) Leptodactylus ventrimaculatus , (16) Aquarana catesbeiana. Photos: Keyko Cruz‐García and Natalia Zapata‐Salvatierra. Guayaquil (Part 3). (17) Barycholos pulcher , (18) Pristimantis achatinus , (19) Caecilia tenuissima . Photos: Keyko Cruz‐García and Natalia Zapata‐Salvatierra. [file ECE3-16-e73504-s002.pdf]

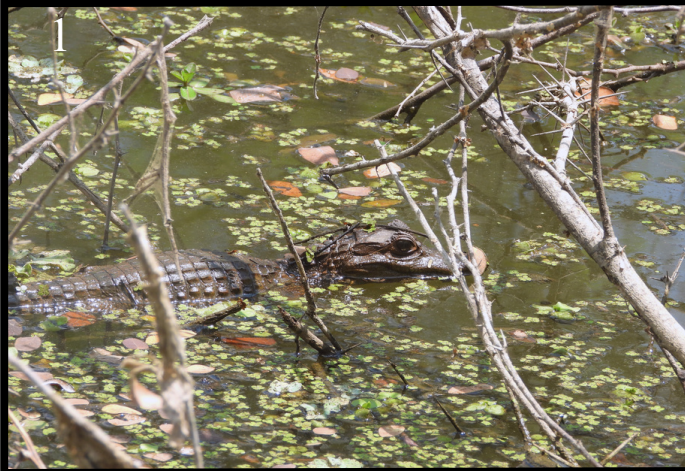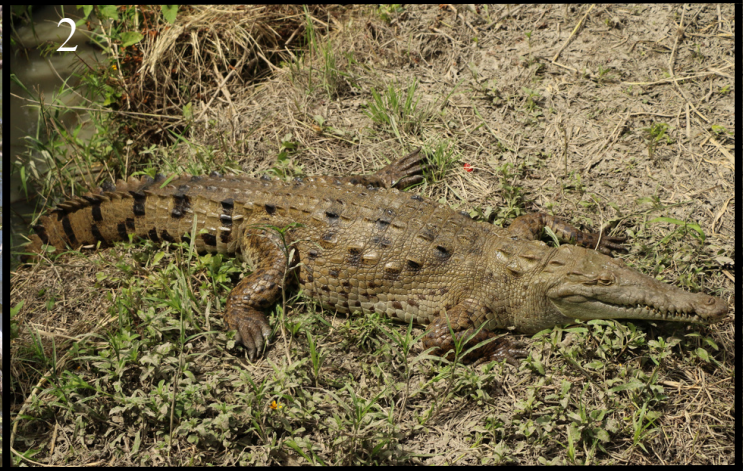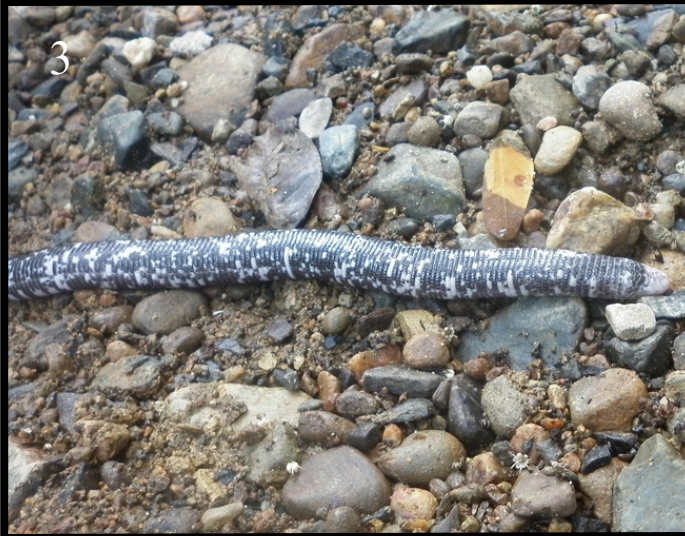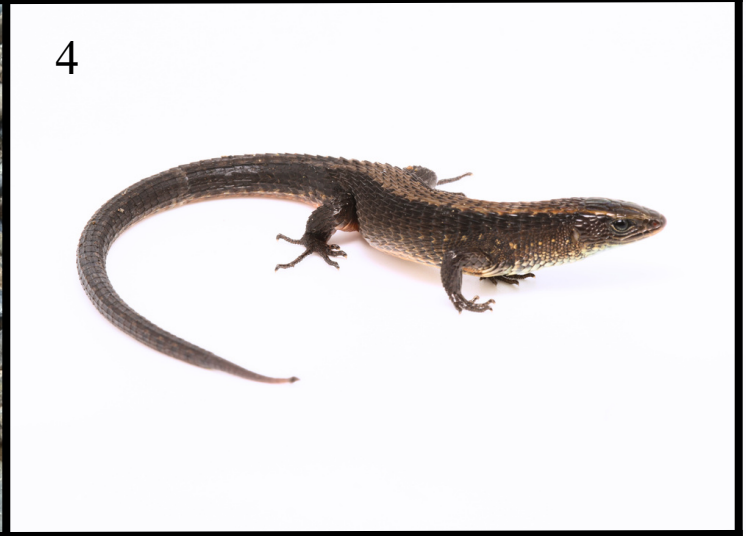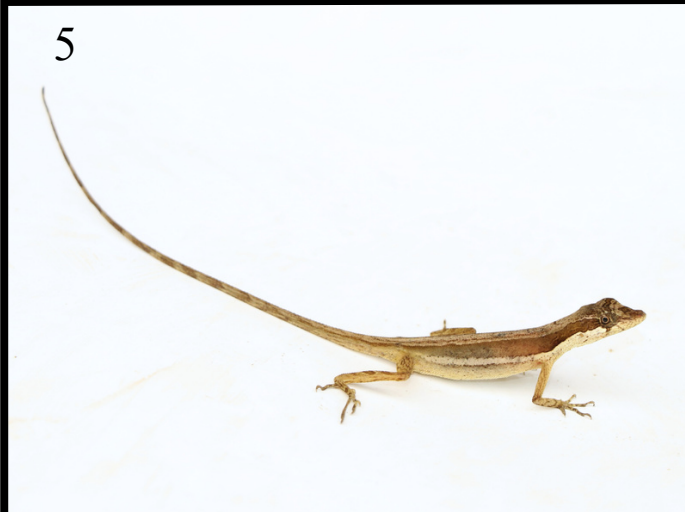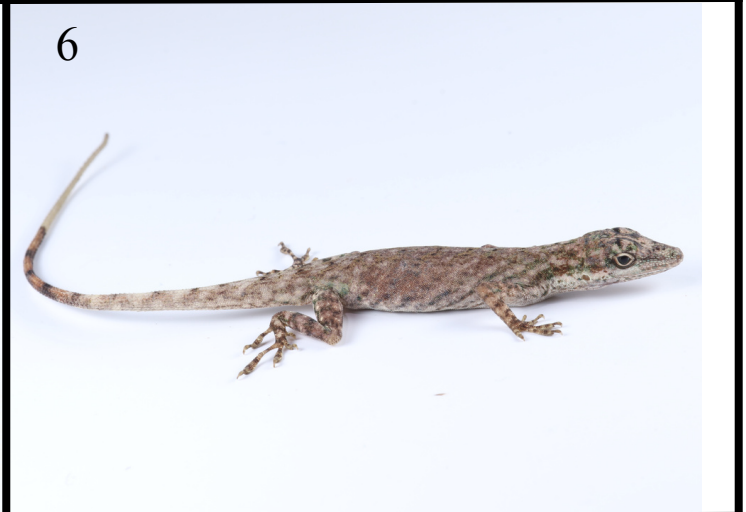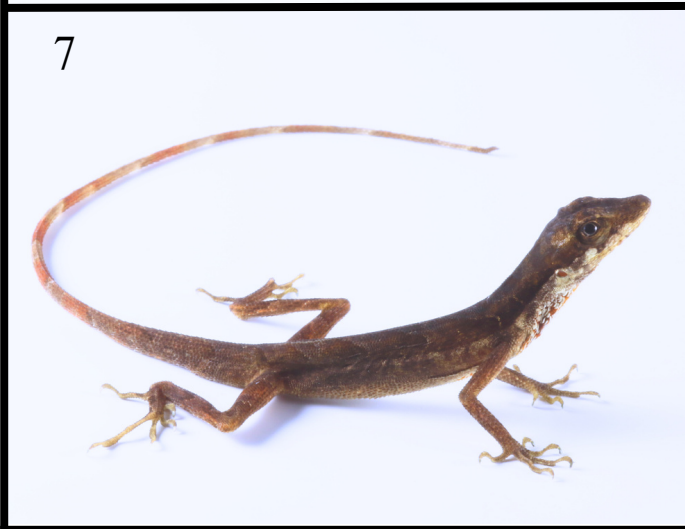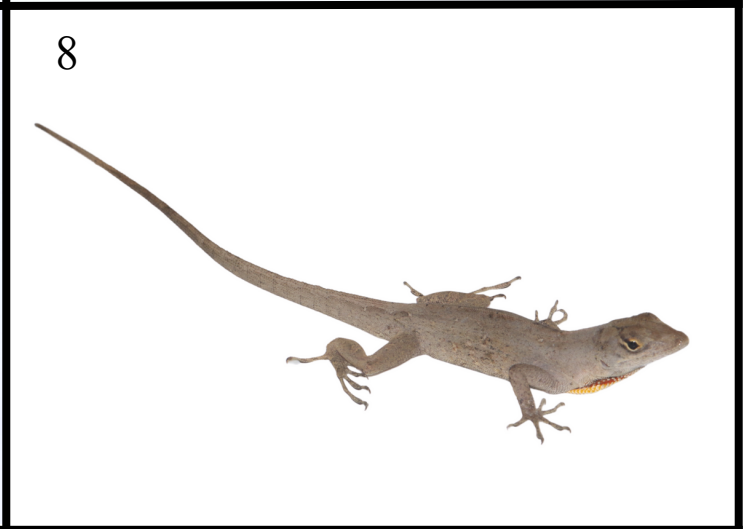

9

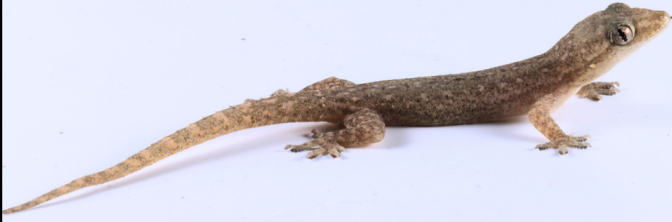

10

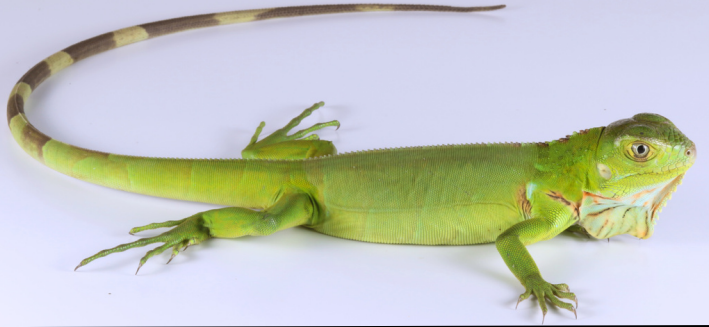

11

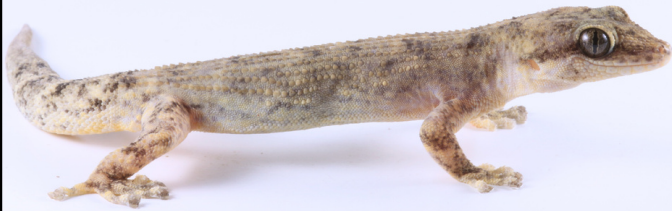

12

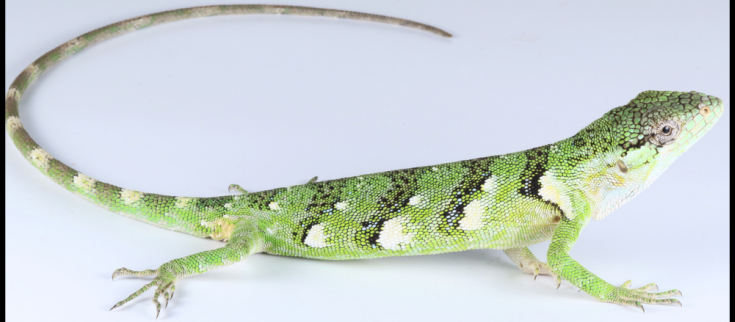

13

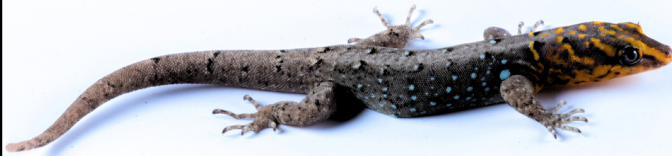

14

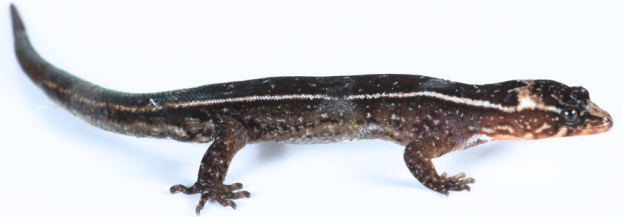

15

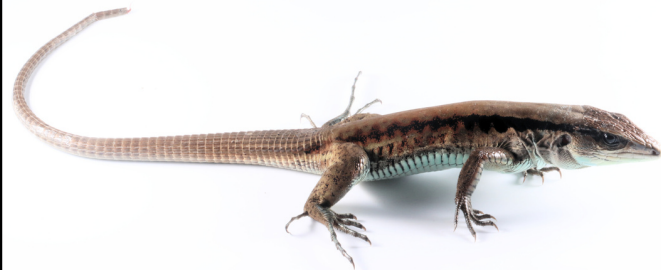

16

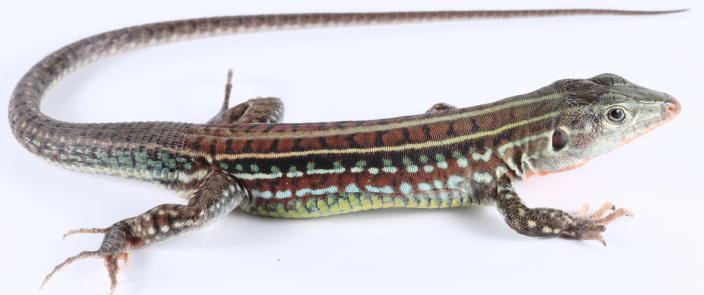

17

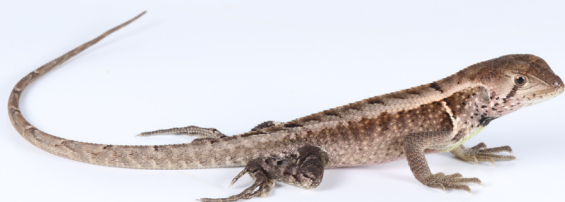

18

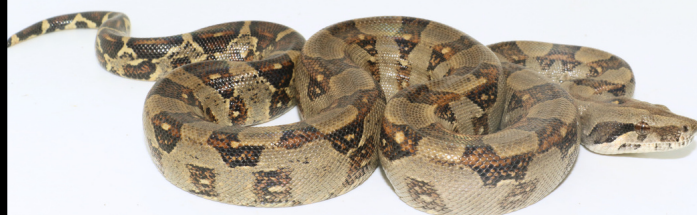

19

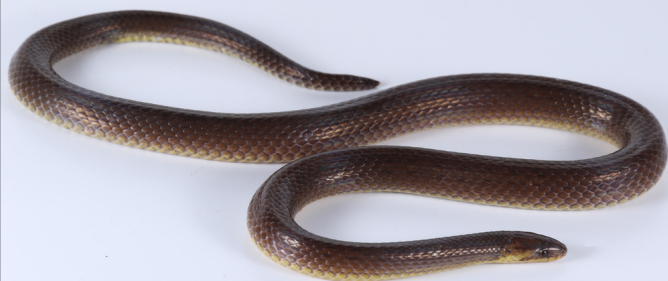

20

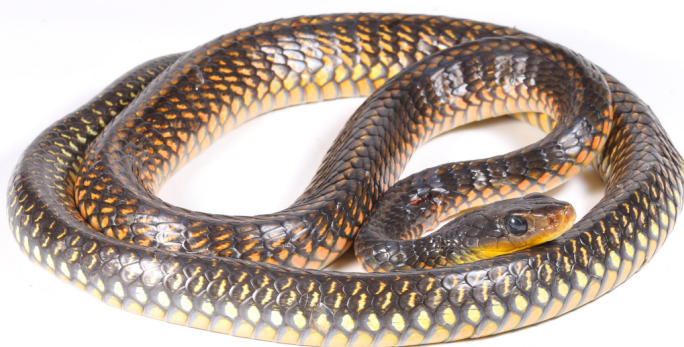

21

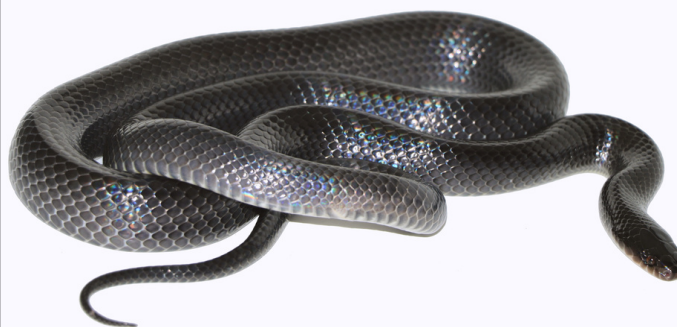

22

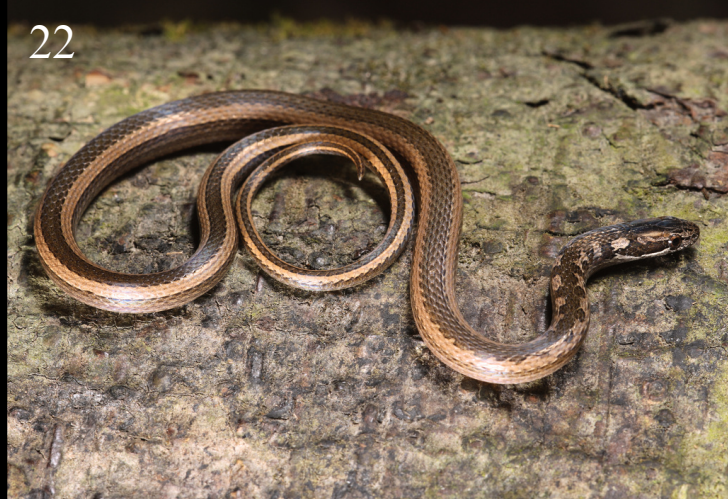

23

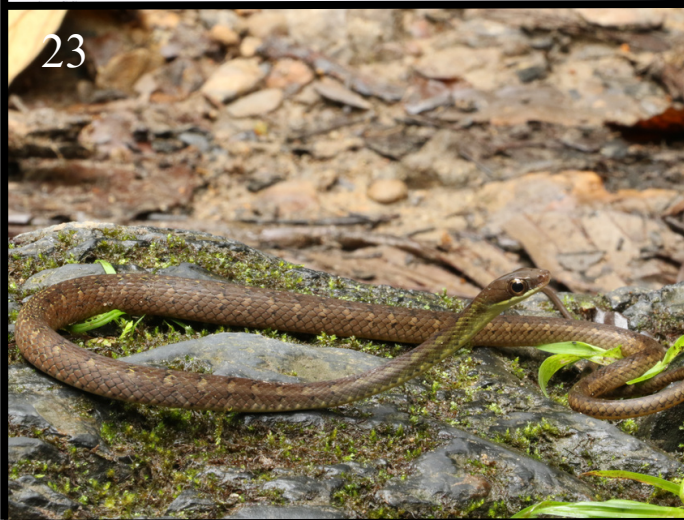

24

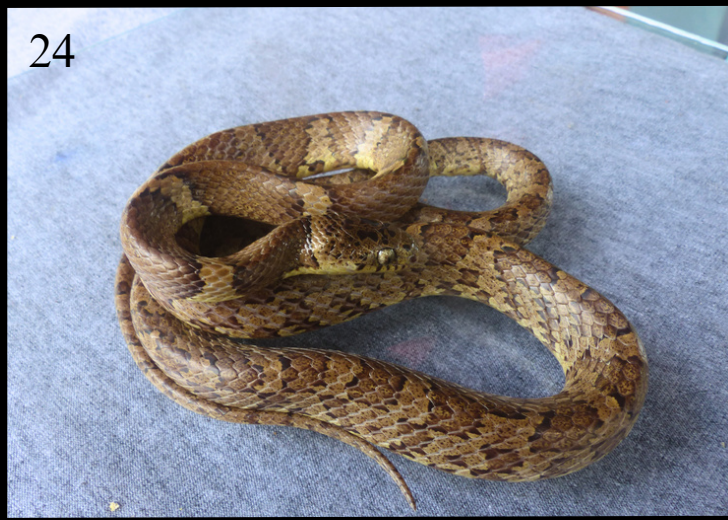

25

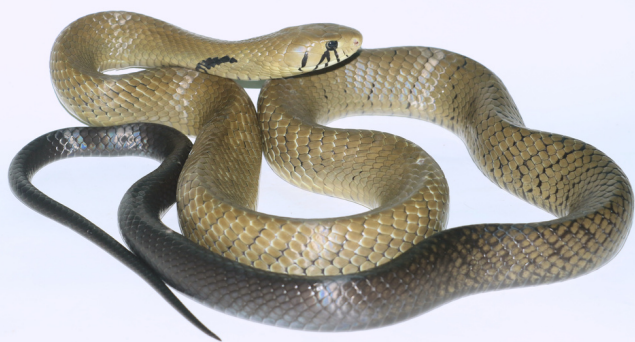

26

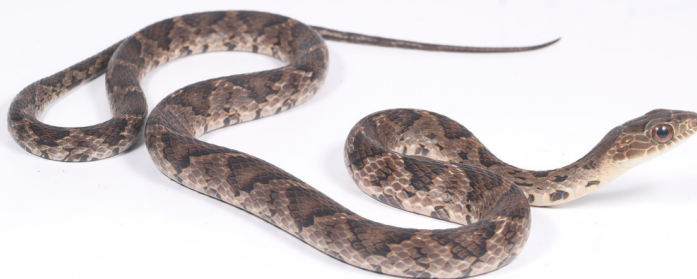

27

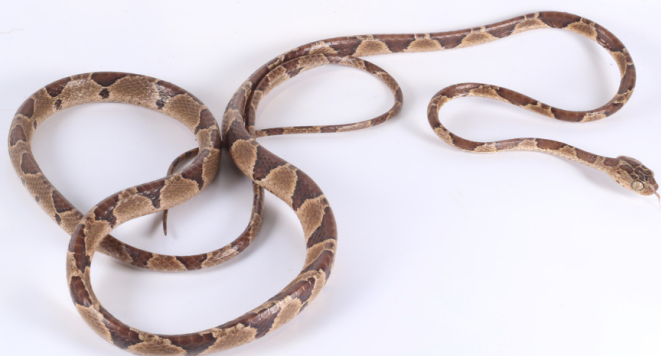

28

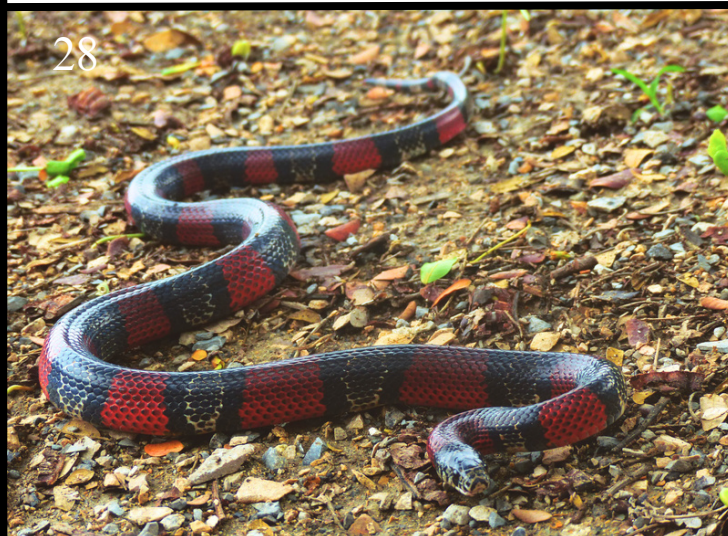

29

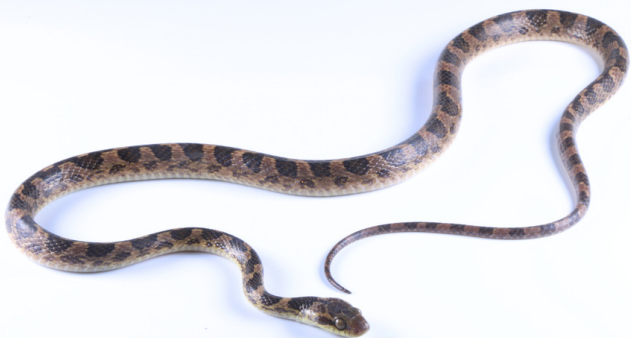

30

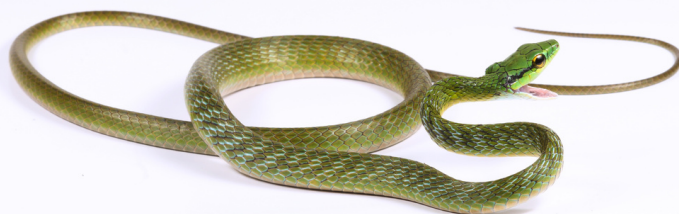

31

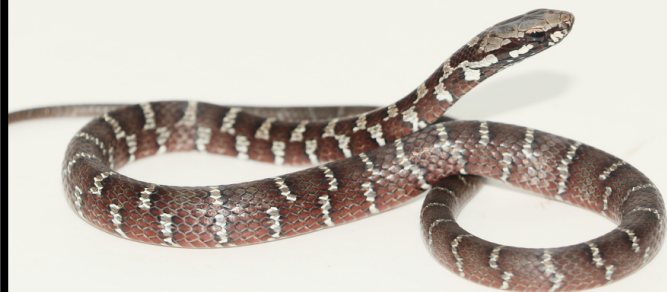

32

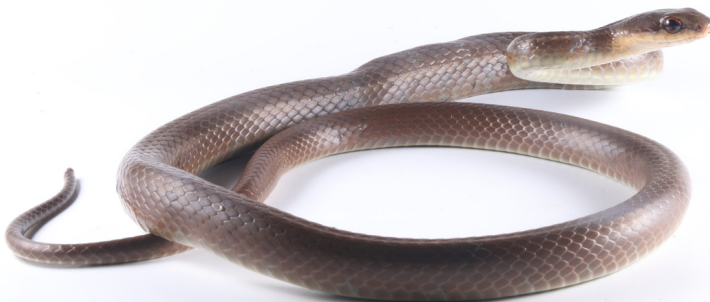

33

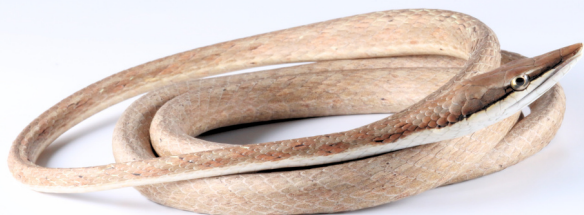

34

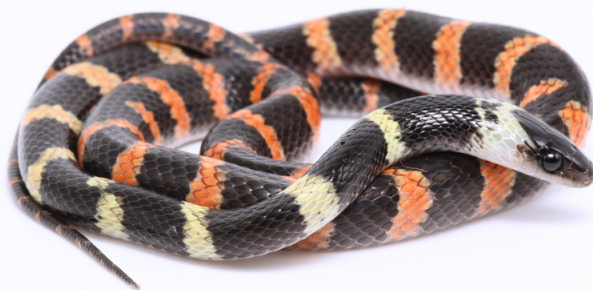

35

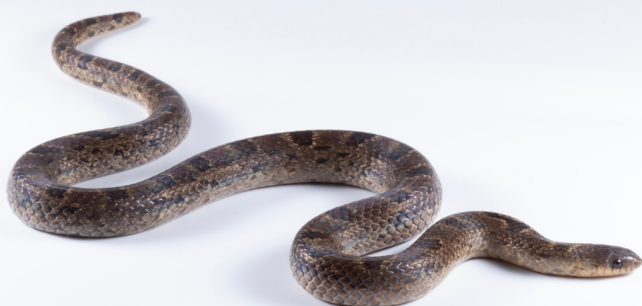

36

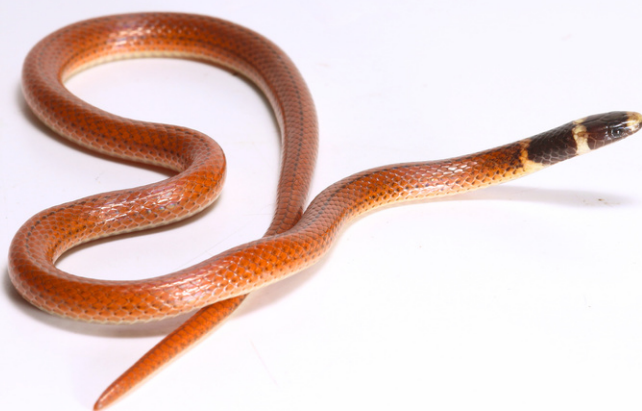

37

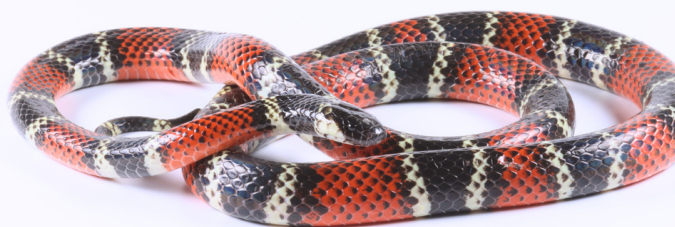

38

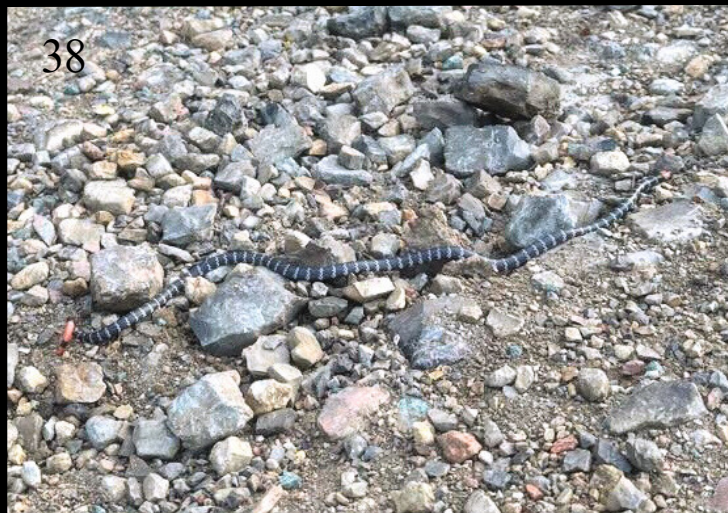

39

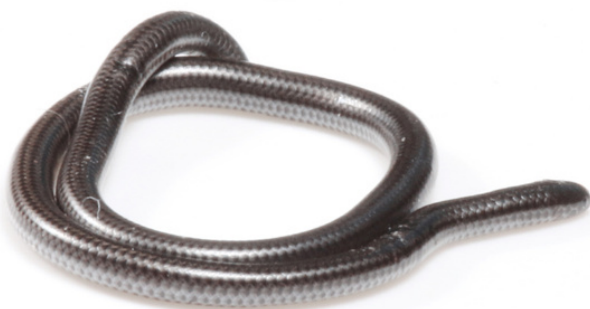

40

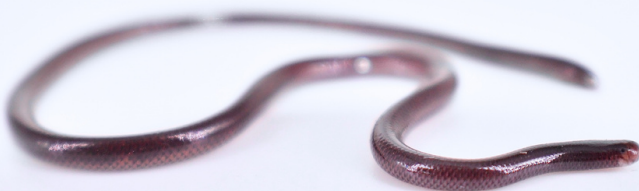

41

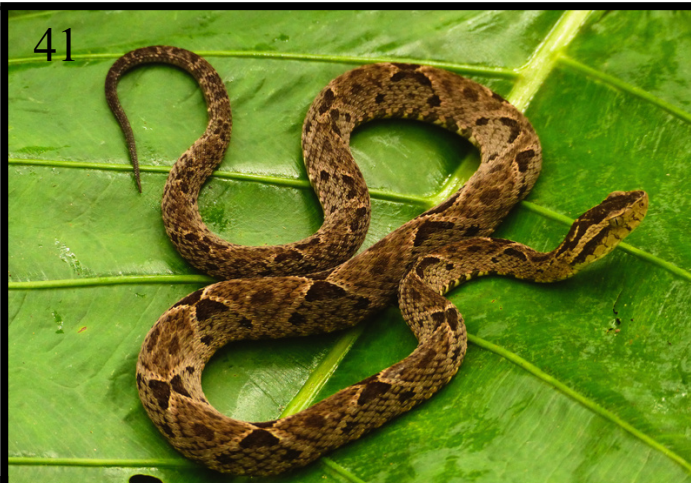

42

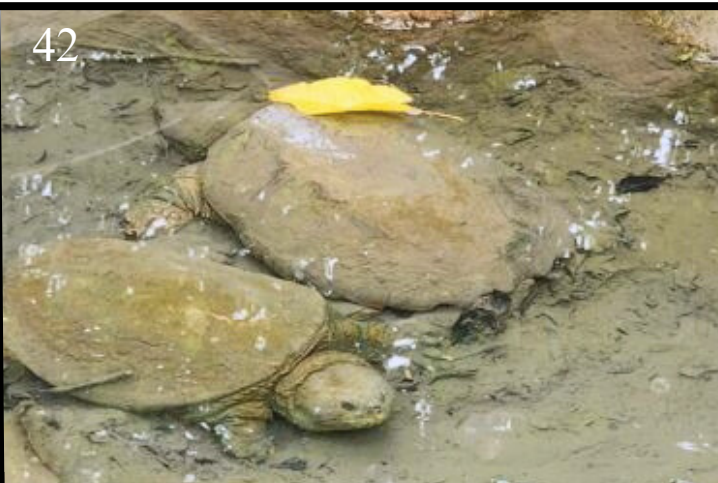

43

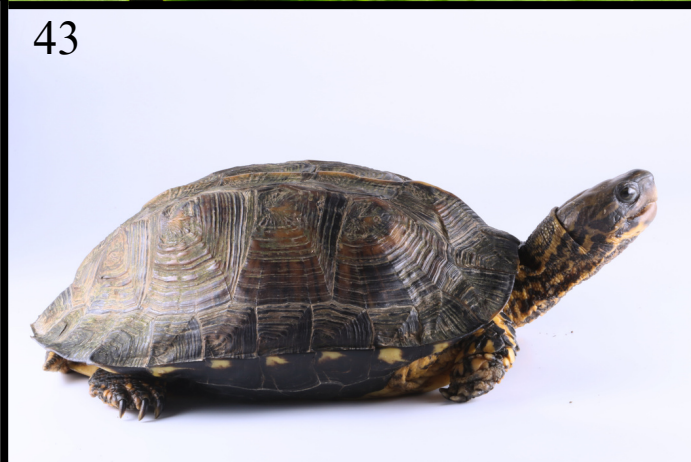

44

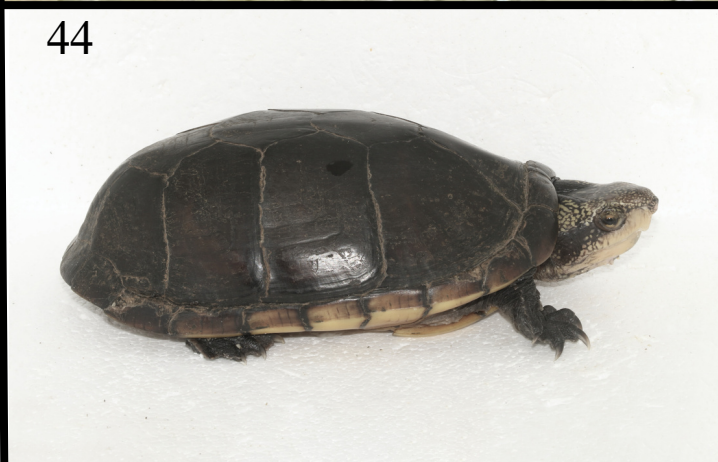

Supplement: Supplementary file 4 — Appendix S4: Photographs of the individuals corresponding to the reptile species recorded in the urban and peri‐urban areas of Guayaquil (Part 1). (1) Caiman crocodilus , (2) Crocodylus acutus , (3) Amphisbaena varia , (4) Alopoglossus festae, (5) Anolis binotatus , (6) Anolis festae , (7) Anolis gracilipes , (8) Anolis sagrei . Photos: Keyko Cruz‐García and Natalia Zapata‐Salvatierra. (Part 2). (9) Hemidactylus frenatus , (10) Iguana iguana , (11) Phyllodactylus reissii , (12) Polychrus femoralis , (13) Gonatodes caudiscutatus , (14) Lepidoblepharis buchwaldi , (15) Holcosus septemlineatus, (16) Medopheos edracanthus. Photos: Keyko Cruz‐García and Natalia Zapata‐Salvatierra. (Part 3). (17) Stenocercus iridescens , (18) Boa imperator , (19) Atractus microrhynchus , (20) Chironius flavopictus , (21) Clelia clelia , (22) Coniophanes dromiciformis , (23) Dendrophidion brunneum , (24) Dipsas georgejetti. Photos: Keyko Cruz‐García and Natalia Zapata‐Salvatierra. (Part 4). (25) Drymarchon melanurus , (26) Drymobius rhombifer , (27) Imantodes cenchoa , (28) Lampropeltis micropholis , (29) Leptodeira ornata, (30) Leptophis occidentalis, (31) Mastigodryas pulchriceps , (32) Mastigodryas reticulatus . Photos: Keyko Cruz‐García and Natalia Zapata‐Salvatierra. (Part 5). (33) Oxybelis transandinus, (34) Oxyrhopus petolarius , (35) Stenorrhina degenhardtii , (36) Tantilla capistrata , (37) Micrurus bocourti , (38) Micrurus mipartitus , (39) Epictia subcrotilla , (40) Indotyphlops braminus . Photos: Keyko Cruz‐García, Melba Morán Soto and Natalia Zapata‐Salvatierra. (Part 6). (41) Bothrops asper , (42) Chelydra acutirostris , (43) Rhinoclemmys annulata , (44) Kinosternon leucostomum . Photos: Keyko Cruz‐García and Leonardo Alava. [file ECE3-16-e73504-s003.pdf]
